# Global droughts connected by linkages between drought hubs

**DOI:** 10.1038/s41467-022-35531-8

**Published:** 2023-01-10

**Authors:** Somnath Mondal, Ashok K. Mishra, Ruby Leung, Benjamin Cook

**Affiliations:** 1 Glenn Department of Civil Engineering, Clemson University, Clemson, SC USA; 2 Atmospheric Sciences and Global Change Division, Pacific Northwest National Laboratory, Richland, WA USA; 3 NASA Goddard Institute for Space Studies, New York, NY USA; 4 Lamont-Doherty Earth Observatory, Columbia University, Palisades, NY USA

**Keywords:** Natural hazards, Hydrology

## Abstract

Quantifying the spatial and interconnected structure of regional to continental scale droughts is one of the unsolved global hydrology problems, which is important for understanding the looming risk of mega-scale droughts and the resulting water and food scarcity and their cascading impact on the worldwide economy. Using a Complex Network analysis, this study explores the topological characteristics of global drought events based on the self-calibrated Palmer Drought Severity Index. Event Synchronization is used to measure the strength of association between the onset of droughts at different spatial locations within the time lag of 1-3 months. The network coefficients derived from the synchronization network indicate a highly heterogeneous connectivity structure underlying global drought events. Drought hotspot regions such as Southern Europe, Northeast Brazil, Australia, and Northwest USA behave as drought hubs that synchronize regionally and with other hubs at inter-continental or even inter-hemispheric scale. This observed affinity among drought hubs is equivalent to the ‘rich-club phenomenon’ in Network Theory, where ‘rich’ nodes (here, drought hubs) are tightly interconnected to form a club, implicating the possibility of simultaneous large-scale droughts over multiple continents.

## Abstract (web summary)

This study shows prominent synchronous co-evolution of drought events in drought hubs in sub-tropical regions, influenced by sea surface temperature patterns and teleconnections. Such simultaneous occurrence of droughts may have detrimental impacts.

## Introduction

Droughts significantly impact water resources, agriculture, energy production, and a host of socio-economic activities^1^. By far, droughts are among the costliest natural disasters^2^. Compared to other hydroclimatic extremes, droughts occur at larger spatiotemporal scales^3,4^, typically spreading over hundreds to thousands of kilometers and often lasting for months to years^1,3^. The impact of drought on different sectors (e.g., water, energy, and agriculture) varies by region and continent depending on the adaptation strategies and resilience of the systems^1^, leading to contrasting biophysical and social consequences^5^.

Recently, spatially compounding droughts have gained prominence due to their detrimental cascading impact on food, water, and energy security^6–8^. Such mega-scale droughts (>10^6^ km^2^) simultaneously occurring across multiple continents can trigger international food price hikes^9^, disruption of trade infrastructure, political instability^9,10^, and even unsound human migration^11,12^. The risk of such synchronous drought conditions across multiple kinds of wheat, maize, and soybean-producing regions has increased in the last four decades^6^. Furthermore, the projected increase in dry spell length and drought frequency^13^ may amplify the possibility of synchronous drought conditions over multiple croplands and pastures^6^. Climate change has already increased drought all over the globe^13,14^. Therefore, understanding the global synchronization (or teleconnection) structure of drought events is critical to quantifying the likelihood of spatially compounding droughts under changing dynamic and thermodynamic conditions. Notably, understanding synchronous drought occurrences over several continents may be the key to regionalizing and quantifying the looming risks of simultaneous bread-basket failures in the future^7^.

Many studies have investigated the role of teleconnections and large-scale circulation in driving synchronous large-scale drought occurrences over different continents^7,15–18^. For example, a few studies have considered the influence of individual climate modes on droughts at regional to continental scale^7^, focusing primarily on atmospheric teleconnections and how they may drive drought conditions. Studies analyzing droughts’ spatial and temporal structure typically use empirical orthogonal function (EOF) or coupled pattern analysis. However, EOFs and related methods lack the ability to explore the complexity of the higher-order statistical interrelationships in climatological data^19^, which is critically important to study the synchronization (teleconnection) of global drought events. Furthermore, previous studies have focused on specific objects, processes, or spatiotemporal scale^20^, without explicit considerations of all possible interactions/dependencies (or covariates)^21^. Typically one or a combination of known teleconnections are selected for analysis to understand their drought-inducing ability. To address these limitations, this study uses a higher-order statistical approach that quantifies and characterizes all possible links of spatial synchrony (of high statistical significance) in the onset of drought events over the whole globe without any assumptions and analyzes the collective behavior of drought onsets. The structure deciphered from data can be used to guide analysis of the physical processes driving the spatial synchrony of drought events, providing crucial information for formulating suitable drought response and mitigation measures.

The evolution of global droughts is controlled by the complex coupled climate system. The relationships between any complex system’s elements (be it in climate, transportation, or other scientific applications) can be represented in terms of complex networks (CN). However, the concept of CN has only been adopted in climate studies recently^3,22–26^. For example, CN-based algorithms have been used to investigate climatic extremes such as extreme summer precipitation^25,27^, heatwave events^28^, and typhoon track detection^29^, along with their complex spatial structures and temporal directionality^25,26,29^. In a recent study, the CN concept was implemented to investigate the spatiotemporal evolution of drought events in the continental US^3^. The results indicate that drought events travel longer distances in the western part of the continental US than in the eastern region. However, CN has not been used in studies related to the synchronization (teleconnections) of global or continental droughts, and the implementation of robust statistical tools to study complex climate systems has been lacking.

Here we investigate the synchronization of global droughts using CN to address the following questions: (a) how are drought events regionally and globally linked to each other? (b) how does the spatial scale of drought synchronization (linkages) vary globally? And (c) how does planetary-scale synchronization occur among drought events, and what physical processes connect the synchronous occurrences of droughts? CN is used to derive higher-order CN measures such as betweenness centrality, which can approximate energy flow or matter in the climatological field. The CN approach allows us to detect the presence of ‘hubs’, ‘bottlenecks’, or ‘core-periphery’ structures in the system, overcoming limitations of EOF and related approaches to provide a better picture of the ‘system’s vulnerability to external perturbations’^19^.

We show that the synchronization structure enabling circumglobal connectivity among drought events is highly heterogeneous (partially scale-free), with a few locations (drought-hubs) affluent in connections. The joint distribution of the network coefficients depicts multiple spatial scales associated with drought hubs and the ingrained core-periphery structure in global drought synchronization. The synchronous global drought events are possibly driven by sea surface temperature (SST) modes (teleconnections) and regional weather systems.

## Results

We employ CN to formulate the synchronization network (see methods) considering the onset of droughts defined by the self-calibrated Palmer Drought Severity Index (ScPDSI) over different spatial locations worldwide. Using the synchronization network, we derive network coefficients to characterize the linkage, scale, and spatially compounding characteristics among drought occurrences on regional to global scales. A detailed definition and mathematical illustration of the network coefficients are presented in Methods, and a brief description and physical interpretation of the network coefficients are summarized in Table 1.

**Table 1:** Complex network-based metrics used in the study and their climatic interpretation

Metric	Network-based description	Climatic interpretation
Synchronization (*Q*)	Likelihood of two nodes oscillating in the same dynamical pattern.	Quantification of concurrence or lagged occurrences of droughts in any two nodes.
Delay (*q*)	The likelihood that event occurrence in one node precedes or succeeds that of another node.	Determines the sequence of occurrence of droughts between any two nodes.
Degree centrality (DC)	A number of connections linked to a node.	A number of grid points displaying synchrony in the occurrence of droughts with respect to a node.
Betweenness centrality (BC)	The influence of a selected node over the transfer of information between other nodes.	The relative proportion of propagation of droughts through the selected node.
Clustering coefficient (CC)	The extent of interconnectivity among the neighbors connected to the node.	The extent of local coherence in drought occurrences among the nodes with respect to a given node.
Mean synchronization distance (MSD)	The weighted mean distance of a node from its connected neighbors. Synchronization associated with the connections is used as a weighting factor.	Mean spatial scale of synchronization (in occurrences of droughts) of the considered node location.
Connection density (*k*_in_)	A number of locations of a considered region connected to a grid location.	A number of nodes experiencing simultaneous drought onset along with the considered node location.
Average synchronization density (ASD)	The magnitude of similar oscillatory characteristics between a region and a considered grid location.	Relative concurrence of drought event onset for a given region and an external node.

### Spatial linkages among global droughts

Degree centrality (DC) signifies the number of grid points displaying synchrony corresponding to an event’s occurrence concerning a given node^27^. In this study, the DC of a grid location indicates the number of neighboring grids (in space and/or time) experiencing simultaneous drought events within the maximum time lag of 1–3 months. The optimal time frame of 3 months is adopted to consider distant spatial co-evolution (connections^3^). Thus, a region with high DC could act as a spatially resonating oscillator^22,23^ that synchronizes with drought occurrences over many other remote parts of the globe within a period of 3 months. In previous climate-network studies, a cluster of high degree^28,30^ temperature and pressure network nodes are named hubs or supernodes^23,24^. Such hubs can modulate the teleconnections of a dynamical system indulging in large-scale communication^22,23^, critical for complex structure and dynamics^31^ of the general circulation of that system.

**Fig. 1:**
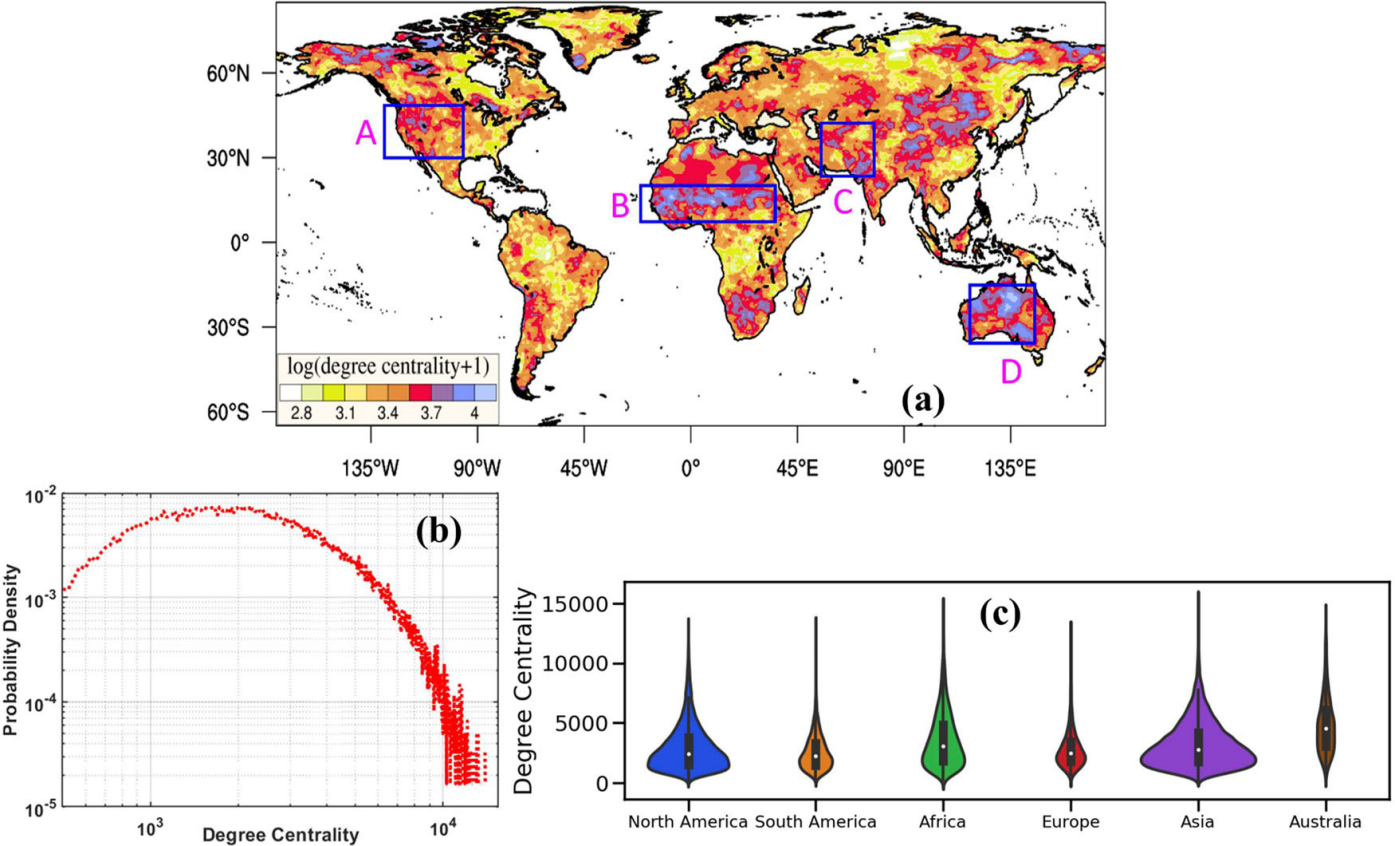
Spatial linkages among global droughts. **a** Degree centrality of the global drought network, Box A-D: regions with high spatial linakge (or degree centrality) **b** degree distribution of the global drought network on a log-log scale with the thick tail characteristics indicating the presence of nodes having very high connectivity (drought hubs), **c** Violin plot of degree centrality for each of the six continents with Australia being the most connected continent.

Globally, DC varies from >100 to more than 10,000 (1/60th to 1/6th of the global area), indicating highly heterogeneous spatial linkages among different regions (Fig. 1a, b). The degree distribution of the drought network (Fig. 1b) and the complementary cumulative distribution function (Supplementary Fig. 1) display strong heavy tail characteristics after an approximate threshold (DC > 5000). This heavy tail of degree distribution is omnipresent over all the continents (Fig. 1c). It implies that a few locations are rich in connections (DC > 5000) and thus possibly synchronize with droughts over other continents. We call these geographical regions drought hubs, which are more likely to synchronize with drought onsets in other regions. Continentally, this is possible if the drought hubs are close to critical climatic features whose variability controls the regional drought dynamics. For example, modulation of Botswana High controls the hydroclimatic extremes in southern Africa^32^. Similarly, through proximity to Northwest USA, the North Pacific High can influence drought conditions in that region^16^. Once triggered by regional temperature and precipitation anomalies induced by climatic features, land-atmosphere processes may control the spatial synchronization of drought by altering the water and energy balance through various mechanisms (e.g., reduced soil moisture and evaporation) and atmospheric circulation^33^.

Land–atmosphere processes are critical drivers for synchronizing drought events within a land region, but they may not support the synchronization of droughts across continents. Instead, the latter is likely primarily associated with large-scale climate indices and associated teleconnection patterns, which can induce atmospheric anomalies such as mid-tropospheric geopotential height globally and cascade into multi-continental drought events. For instance, El Niño SST condition in the tropical Pacific strengthens the pressure ridge over the northwestern USA through Rossby waves^16^. This may, in turn, induce amplifying drought conditions over Northwest USA in the subsequent spring or summer, as already noted in previous studies^16,34^. Although drought synchronization across continents may be primarily driven by modes of climate variability involving the coupled atmosphere–ocean system, recent studies have also demonstrated the long-range impacts of soil moisture anomalies that induce Rossby waves^35^, similar to Rossby waves induced by the El Niño SST anomalies. Whether and how land-induced teleconnections may contribute to drought synchronization across different continents requires further investigation.

Here, we hypothesize that drought hubs are observed due to their proximity to regional climate modulators or features and their ability to interact with global teleconnections (e.g., El Niño). Interestingly, the climatological locations of subtropical anticyclones such as North Pacific High, Botswana High, and the Tibetan High display increased DC (Fig. 1a), supporting their role as drought hubs. Noticeably, North American areas with high DC (DC > 5000) are in the western US, particularly Northwest and Southwest US. Such high DC over the western US (Box A, Fig. 1a) is possibly due to the stationary upper-tropospheric ridge over the Rockies and the climatological location of North Pacific High. As mentioned earlier, these climate features strongly respond to anomalous SST anomalies^16^, especially in the Pacific Ocean. Furthermore, northward displacement of the midlatitude storm track (due to La Niña) suppresses frontal activities in Michigan Lake and the Midwest region. Thus, displacement of the storm track may initiate drought over the Midwest (high DC), triggering the famous North American droughts over the 20th Century^17,36^. Compared to the Pacific, a relatively lower degree of centrality over eastern North America indicates the smaller global influence of the Atlantic SST anomalies that primarily influence droughts over the eastern US^37^ and the Southern Great Plains. The low DC in the eastern US can also be due to a lack of precipitation seasonality. Precipitation in this region is more evenly distributed across the year, making it relatively easier to break out of drought, compared to a region such as the western US with more pronounced dry/wet seasons.

A band of very high DC values is also identified over the Sahel region (Box B, Fig. 1a), extending from the continent’s west to equatorial eastern Africa. This pattern of high DC values is possibly a sign of the continental-scale Sahel droughts in the twentieth century^38,39^. These drought events are influenced by large-scale forcing such as the warming of tropical oceans, especially the Pacific and Indian Oceans, superimposed on amplified warming of the Southern Hemisphere oceans compared to the Northern Hemisphere (interhemispheric temperature gradient), most evident in the Atlantic^38^. High DC values are also present over the continent’s south, extending from Zambia and Namibia to South Africa. Such high DC possibly indicates the influence of El Niño-induced Rossby waves on the stationary upper tropospheric pressure ridge, Botswana High^32^, typically triggering drought conditions through reduced precipitation and increased temperature^32^. However, regional oceanic and atmospheric anomalies (i.e., southwestern Indian Ocean SST) may also play a role^38^.

Over Europe, large-scale forcing such as Atlantic and Pacific SST affect the spatial distribution and thus control the possible spatial linkages among them^40^. High DC locations are present over the Alps Mountain and the Iberian Peninsula in the south and southwest. In the north, the high DC values are prominent in the Scandinavian Peninsula and Northeast Europe. The dipolar concentration of increased spatial linkages in the north and south of the European continent possibly shows the linear influence^41^ of the North Atlantic Oscillation (NAO), defined as the difference of atmospheric pressure at sea level (SLP) between the Icelandic Low and the Azores High^42^. Other teleconnections such as ENSO, pacific decadal oscillation (PDO), and atlantic meridional oscillation (AMO) also substantially influence the spatial distribution of droughts over Europe. For example, the positive (negative) mode of NAO typically increases drought occurrences over the South and Southwest (Northwest) of Europe through northward migration of the North Atlantic jet stream^41^. On the other hand, cold phases of ENSO and PDO also can force drying conditions over Southern and Southeast Europe^40^.

Topography plays a substantial role in regional to large-scale connectivity in nearly all climate extremes^27,43^. In the case of drought, the spatial distribution of DC indicates the potential influence of topography (Supplementary Fig. 2a, b), as most regions with high DC values situate across areas with higher elevation, irrespective of the continents. For instance, the north–south orientation of the America Cordilleras forms a physical barrier confining the Great Plains low-level jet and South American Monsoon jet to the east of the mountain ranges. Thus, topographic features modulate the propagation of regional rainfall events, and consequently, droughts across the Cordilleras display spatial synchrony with many locations (Fig. 1a). Similar spatial distribution is distinct over the Amazonian basin, where upstream areas of the Amazon River adjacent to the eastern slope of the Andes display an increased number of spatial connections (DC > 5500) compared to its downstream regions. However, the relationship between topography and network characteristics may not be linear, as the evolution of droughts is controlled by a combination of climate and catchment characteristics^44^. To ascertain the role of topography, the kernel density distributions for DC values of grid locations corresponding to different elevation bands at 1000 m intervals are shown in Supplementary Fig. 2c. Noticeably the median value of DC increases towards higher elevation, so more locations with higher elevation are likely to display more synchrony in drought onset than low elevation regions.

Although the proximity of high DC values and higher elevation possibly indicates the control of topography on precipitation regimes over Asia, the high DC also suggests the modulation of the dominant regional dynamics by oceanic forcing. For example, in Asia, high DC regions are evident in Central and Southwest Asia (Box C, Fig. 1a), one of the world’s driest places. Latent heat release of convection over the tropical eastern Indian Ocean triggers westward propagating Rossby wave packets manifesting as anticyclones that suppress cyclonic activity over Central and Southwest Asia (high DC) through anomalous subsidence^45^. Furthermore, the warm western Pacific basin during La Niña also strongly enhances this regional dynamics^45^. In contrast, areas with high DC over Tibet (climatological location of Tibetan High) and Southeast Asia possibly imply the influence of El Niño. During the warm eastern Pacific (El Niño), an eastward shift in the zonal Walker circulation and anomalous fluctuation of the inter-tropical convergence zone over the Indian Ocean favor subsidence that suppresses Indian and Southeast Asian monsoon. High DC regions over Northern China possibly indicate the influence of local (i.e., Pacific-Japan) and global (i.e., ENSO) teleconnections on East Asian monsoon.

Over Australia, high DC locations are evident north and west of the continent (Box D, Fig. 1a), extending to the Murray-Darling basin in the southeast. Interestingly, high DC locations over Northern Australia are also the climatological location of the upper tropospheric ridge, Bilybara High, responsible for bringing moisture during the Australian summer monsoon^32^. Drought over such a high DC region indicates a spatially extensive negative precipitation anomaly during the Australian monsoon. High DC areas over southeast Australia imply the control of El Niño in inducing droughts over these regions. The well-known Millennium drought (2001–2009), centered over the Murray–Darling basin, has been attributed to El Niño and the intensification of the subtropical ridge in Southern Australia^46^.

### Robustness of the spatial linkage of droughts

To test the robustness of the results, we compared drought characteristics during two non-overlapping time periods, 1901–1958 and 1959–2015, to evaluate whether the spatial structure of drought synchronization may depend on the period of analysis. The drought hubs based on the entire period are also observed in the two non-overlapping periods (Supplementary Fig. 3a, b). For example, the DC characteristics over the prominent drought–hub regions such as Northwest USA, southwest South America, South Africa, and Australia remain similar in magnitude and spatial extent when the original study period is segregated into two non-overlapping periods. However, the spatial extent of drought synchronization increased during the second period (1959–2015) over the Sahel, Eastern Asia, and Southern Europe. The large-scale drought synchronization over these regions during 1959–2015 is likely associated with continental-scale drought events during the second half of the 20th century, such as the 1982–1986 African Drought, 1975–1976 and 2003 European Drought, 1984–1988 and 1997–1998 Asian drought^47^. Such continental-scale drought events may lead to increased spatial synchrony in drought for these regions.

To evaluate the sensitivity of our results to the variables used to define droughts, we compared the spatial linkage of droughts based on soil moisture drought indicator and ScPDSI for the time period of 1959–2010. The ERA-20C data is available up to 2010, and it provides a better representation of meteorological (i.e., precipitation) variables^48^ and drought^49^ among the 20th-century reanalysis datasets. First, we identified drought events when the soil moisture is less than 15% of the climatological soil moisture value for a particular month^4^. The 15th percentile was chosen to approximate the moderate drought that is considered in the current study with respect to ScPDSI^4^. The similar spatial pattern between the DC for the soil moisture drought (Supplementary Fig. 4b) and ScPDSI (Supplementary Fig. 4a) indicates the robustness of the spatial network patterns of drought during the selected time period. For instance, DC characteristics over drought-hub regions such as the Sahel region, south of Africa, Australia, and Northwest USA were similarly based on ERA20C soil moisture drought and ScPDSI. However, some discrepancy mainly exists in the eastern USA, southeastern Asia, and southeast Africa, where soil moisture drought displays a higher degree of centrality. As drought characteristics (e.g., severity and duration) vary between drought indicators as well as the thresholds for different drought categories (e.g., moderate and extreme droughts), variations in the spatial synchronization of drought are expected^3^.

The robustness of maximum lag time is evaluated based on DC obtained from 3- and 6-month lag times. The latter enables us to incorporate the spatially synchronized drought onsets over consecutive seasons. The spatial distribution of DC (Supplementary Fig. 5a) and the scatterplot of DC with maximum time lags of 6 months vs. 3 months (Supplementary Fig. 5b) show that the spatial distribution of DC is not sensitive to the lag times, although the magnitude of linkage substantially increases with the time lag. The latter is self-evident since increasing the value of maximum time delay would allow more extreme events for each pairwise estimate of drought synchronization to be included.

### Regional and global topology

DC characterizes network connectivity concerning each grid location, but it does not describe the role of an area in regional connectivity or how a grid location influences the regional or global topology. We present betweenness centrality (BC) and clustering centrality (CC) to quantify the latter. BC represents the relative amount of global information passing through a CN system’s specific node^24,50^. A spatial domain with a higher magnitude of BC is likely to considerably influence the large-scale synchronization of drought events by controlling the flow of information (e.g., the deficit in moisture flux) between any two locations. On the other hand, CC captures the extent of interconnectivity among the neighbors connected to the node^27^. Therefore, drought events are more likely to be spatially connected in a region of high CC, and thus the region has a limited possibility of large-scale synchronization^27^.

**Fig. 2:**
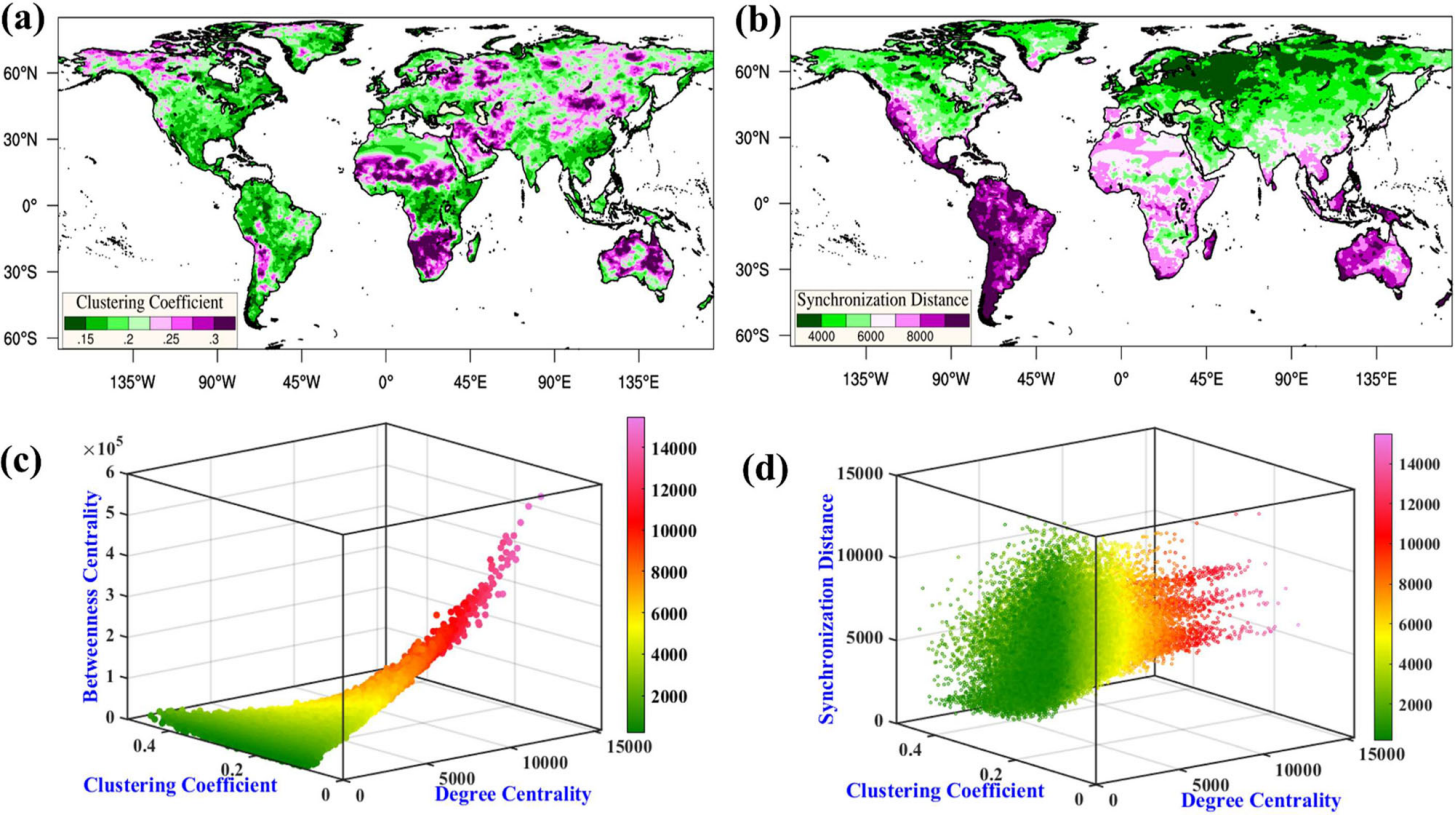
Regional topology & spatial scale of synchronization of global drought events. **a** Clustering coefficient (CC), **b** Mean synchronization distance (MSD) of the global drought network, JOint distribution of **c** degree centrality (DC), betweenness centrality (BC), clustering coefficient (CC), and **d** degree centrality (DC), clustering coefficient (CC) and mean synchronization distance. Red scatter points indicate low CC, high BC (**c**) and multiple spatial scales of synchronization (MSD, **d**) associated with drought hubs (high DC). The colormaps in both **c**, **d** carry the value of degree centrality.

Global spatial plots of BC and CC are provided in Supplementary Fig. 6 and Fig. 2a, respectively. A robust spatial correlation (*ρ* = 0.83) exists between DC (Fig. 1a) and BC (Supplementary Fig. 6), implying that drought hubs (high DC) play a central and decisive role (high BC) in the topology of the complex drought system. Drought events are synchronized in a core–periphery structure where high-degree nodes occupy the central (or core) locations in regional/continental drought systems. In CNs, ‘core–periphery’ refers to the presence of mesoscale structures (similar to communities) consisting of loosely connected peripheral nodes (low DC) surrounding densely connected core nodes (comparable to the design of star networks) with high DC. Furthermore, the high correlation between DC and BC indicates that the drought hubs in a particular region/continent synchronize regionally and with other drought hubs over different continents, thus controlling the possible teleconnection (synchronous) among distant regions. Therefore, calling such clusters of nodes with high DC and BC hubs or supernodes would be no misnomer^23^. Furthermore, the hubs constitute the network’s resilience^22,50^, possibly implying that local or random drought mitigation efforts would not be able to mitigate (or stop) worldwide natural drought occurrences effectively.

Once DC and BC are employed to identify the hubs (high DC, high BC) of the network, we use CC to measure the local spatial connection density (LSCD) of the drought network^25,26^. The significance of LSCD is multifold. A high value of LSCD primarily indicates spatial continuity of the regional drought events. Furthermore, combined with low DC (low BC), high CC value locations possibly act as peripheral nodes. Discerning the peripheral nodes is imperative as they typically decide the spatial limit of the regional/continental drought systems centered around the drought hubs. To distinguish the peripheral nodes, we provide the three-dimensional distribution of DC, BC, and CC (Fig. 2c). A triangular distribution with grid locations having low DC and low BC mainly concentrated around high CC values is evident. In contrast, the probability of high CC decreases with increased DC and BC, indicating drought hubs primarily have low CC values. Exceptions are the Sahel and South Africa (high DC, high BC), where high CC values suggest that drought events are typically spatially contiguous. The observed twentieth-century drying trend over both of these regions^51^ and the higher likelihood of spatially synchronous drought conditions over the Sahel and South Africa may explain the episodes of large-scale droughts observed over these areas^38^. Comparatively high CC values (>0.3) are present in desert regions and regions sandwiched between high topographies (in Australia), showing that drought events over these regions are spatially continuous and limited. Globally, peripheral nodes (low DC, low BC, and high CC) primarily surround the drought hubs (i.e., Northeast Brazil) or situate between them (i.e., Northeast Australia).

### Spatial scale of synchronization

The spatial scale of synchronization of drought events is quantified by the mean synchronization distance (MSD). MSD refers to the average geographical distance beyond which a grid location does not display any significant synchronization in the occurrences of drought events. In the current context, we calculate the MSD of each grid location concerning its all-global connections. Therefore, MSD indicates the mean geographical distance of a grid location’s influence (teleconnection) on drought occurrences over the same or other continents.

MSD varies between 2000 and 10,000 km, indicating regional to circumglobal connections among drought occurrences worldwide. Globally, MSD increases from the northern to the southern hemisphere (Supplementary Fig. 7a), probably due to the higher geographical distance among the continents in the southern hemisphere compared to the north. This could lead to the probability that droughts over the southern hemisphere are synchronized over more considerable distances than their northern counterparts. Furthermore, droughts over coastal regions have a much larger MSD than interior regions, and this is prominent irrespective of the continents or the hemispheres (Fig. 2b). We also present a three-dimensional scatterplot of DC, CC, and MSD (Fig. 2d) to distinguish the dependency between these structural quantifications (network coefficients) of the global drought network, showing multiple geographical scales in MSD associated with the drought hubs (Fig. 2d). To clarify the stratified spatial scales, we also provide a joint distribution of DC and MSD in Supplementary Fig. 8.

Over North America, droughts in the western US display spatial synchrony over a larger spatial scale than in the eastern half of the continent. A subtle difference of 3000 km in scale indicates a more substantial large-scale influence on droughts over the western and northwestern US. This zonally varying MSD over the USA has been reported in previous works^26^, although the quantified scale may be comparably smaller because of the regional nature of the study^3^. MSD is also substantially higher over the northeastern US and Central America, indicating possible large-scale influences such as NAO and ENSO, respectively. Over South America, high MSD is most prominent over the Andes, clearly portraying the effect of topography on large-scale divergence and convergence of moisture in the continent^26,43^. High MSD is also evident over the Amazonian basin, Northeast Brazil, and the Bolivian High (Altiplano) regions.

In Africa, areas around the Congo basin sandwiched between the Sahel and South Africa display high MSD and low CC despite having comparatively fewer spatial linkages (low DC and low BC). This possibly indicates that those areas act as a bridge connecting drought events in the Sahel and Western Africa^4^. For example, spatial drought propagation from West and Central Africa to the Sahel region is documented in the previous studies^4,52^. Such spatial propagation also establishes a lag-synchronization among the considered regions. In addition, large-scale oceanic (Atlantic, Pacific, and Indian Ocean) influence is prominent in the West, east, and South African continents, validating the observed high MSD in the respective regions. Interestingly, comparably low MSD surrounds Lake Chad in the north and Lake Nyasa (along with Zambezi River) in the south. Such large waterbodies imply a possible dependence of the surrounding region on the local convective rainfall and thus possibly less influence on local precipitation dynamics by the large-scale circulation.

Over Europe, like DC and BC, MSD is higher in the Scandinavian region in the northwest and the Iberian Peninsula in the south. These two regions are typically influenced by different modes of NAO^41^, which also synchronize droughts over a large part of eastern North America and north, east, and west of Asia. The synchronization scale strongly increases south of the Indian subcontinent and Southeast Asia. It is possibly due to the more substantial influence of anomalous tropical heating (i.e., ENSO, Madden–Julian Oscillation (MJO), and Indian ocean dipole) on droughts over these regions, as documented in previous studies^45,53^. MSD over Australia varies from 4000 km to 10,000 km, with the lowest scale present over Queensland (also high CC, low DC, low BC), possibly as this region is sandwiched between higher topographies both east and west. The highest values of MSD are present north and southeast of the continent. These areas are the climatological locations of the upper-tropospheric subtropical anticyclone (Bilybara High) and lower tropospheric subtropical ridge. These atmospheric features have a strong influence on rainfall variability and thus droughts over the Australian continent.

### A rich club phenomenon in southern hemisphere droughts

**Fig. 3:**
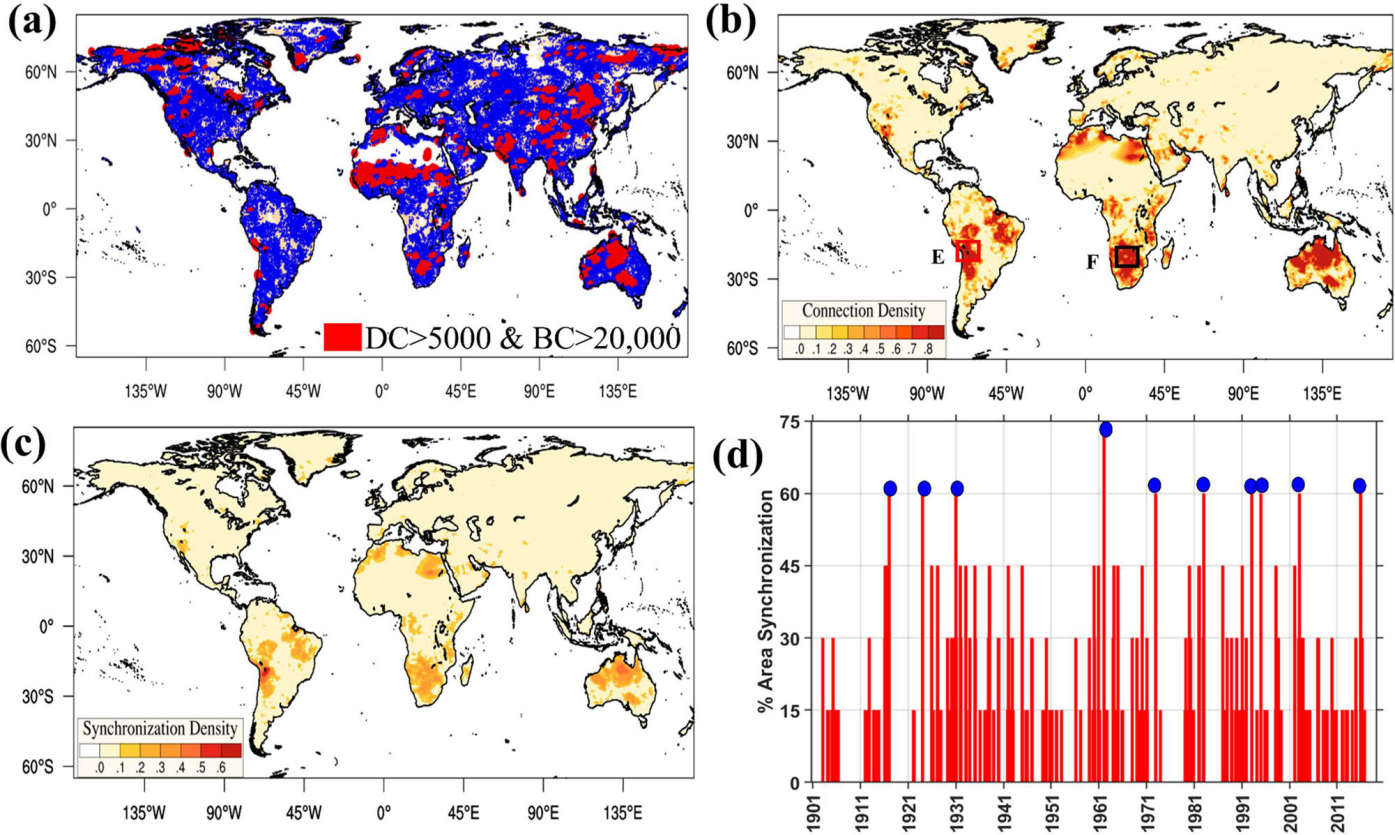
A rich club phenomenon of drought events in southern hemisphere. **a** Drought hubs (degree centrality > 5000 and betweenness centrality > 20,000) are plotted in red, **b** connection density, **c** average synchronization of region chosen near altiplano (climatological location of Bolivian high) over the whole globe. The region is chosen for demonstration for its high connectivity (degree centrality > 8000) and the highest spatial scale (mean synchronization distance > 10,000 km) **d** minimum of percentage area synchronization of both region C and region D over Altiplano and South of Africa. The blue dots indicate the months when drought condition is dominant over both Box E and F (**b**).

Earlier, we illustrated that the DC distribution of the global drought network displays a heavy tail (Fig. 1a, Supplementary Fig. 1) after an approximate threshold (DC > 5000). This implies that a few locations are affluent in spatial linkages (Fig. 3a) and typically form the network’s central core. In Network Theory, nodes (here, grid locations) imparting such robustness by intertwining many connections are called hubs^24,54,55^ (here, drought hubs). Furthermore, a strong spatial correlation between DC and BC indicates the affinity among the drought hubs. This tendency of high-degree nodes (here, drought hubs) to connect with high-degree nodes is called homophily^56^ (the tendency of a location to link with other areas of similar degree of connectivity, see Methods). For global drought, such affinity among drought hubs renders synchronous occurrences of regional droughts over several continents, possibly leading to simultaneous bread-basket failures^6^. A notable example is the 1982–1983 El Niño induced spatially compounding drought over multiple continents^17,36,57^. Furthermore, climate modes such as NAO, PDO, and AMO individually or in combinations may force episodes of spatially compounding (synchronous) droughts in their respective influence regions.

To ascertain homophily, we employ two network coefficients: The assortativity coefficient (AC) (Methods) and rich club metric^58^. The AC quantifies the tendency of the network nodes to primarily link with nodes of similar connectivity, implying that high-degree nodes would link more often with other high-degree nodes. The rich club metric is designed to measure the extent to which well-connected nodes (hubs) also connect to each other. The estimated positive value of the assortative coefficient (0.16) confirms the homophilic characteristics among drought hubs. On the other hand, the monotonically increasing value of the Rich club metric (Supplementary Fig. 9) indicates that the highest connectivity is among the high-degree nodes. Such synchronous behavior of drought-hubs may be referred to as the ‘rich club phenomenon’, already observed in other networked systems^59^. Hereafter, we use CN analysis to illustrate the affinity among the drought hubs through synchronous drought events in the subtropics and midlatitudes of the southern hemisphere. Furthermore, we calculate the composite of precipitation and SST anomaly corresponding to the synchronous period (of drought occurrences) to pinpoint the physical mechanism triggering such homophilic characteristics.

### Synchronous occurrence of large-scale drought events in the Southern hemisphere subtropics

We focus on Altiplano, Bolivia (climatological location of the Bolivian high) to illustrate the Rich club phenomenon or the affinity among drought hubs. The upper-level anticyclone, Bolivian High, forms in response to condensational heating over the Amazon. Including the Botswana High and Bilybara High, this stationary climate feature is also part of the subtropical anticyclonic systems in the southern hemisphere connected through a longwave regime^60^. Such long-distance connections among the climate feature further validate the observed high MSD over the southern hemisphere and play a vital role in upper tropospheric circulations during Austral summer. Areas near Altiplano display high DC, high BC, low CC, and maximum MSD, strengthening the assertion of planetary-scale synchronization (Supplementary Figs. 8 and 10). We select Box E in Fig. 3b with the size of 1.5° × 1.5° consisting of 9 grid locations (total area = 22,500 km^2^) based on values of the connectivity (DC > 8000) and the spatial scale (MSD > 10,000 km). Unlike other studies, this comparatively small area is more probable to synchronize with a sizeable global area in drought onset (DC > 8000, MSD > 10,000 km). Corresponding to the desired region, we calculate the CD (Methods) and average synchronization density of all grid locations (Methods). A high value of both quantifies the relative number and strength of the connections shared by other grid locations within the considered region.

Over South America, the CD (along with average synchronization density) is the strongest in Northeast Brazil (Fig. 3b, c), indicating synchronous drought events over the Altiplano (in 1–3 months) when drought is prominent over Northeast Brazil. High CD and high average synchronization are noticeable over North and South Africa, southwest Asia, and Northern Australia. Interestingly, all these regions display high DC, high BC, and high MSD, further validating the affinity among the drought hubs. Such simultaneous (or lagged) concurrence has been reported in previous studies^60,61^. On closer look, one pattern is evident. For example, areas with high CD in South Africa and Northern Australia are the typical influence region of the Botswana High and Bilybara High^32^. Along with the Bolivian High, these upper tropospheric stationary anticyclones play a vital role in the precipitation regimes over these continents^32,60^. Thus, simultaneous influence on these stationary ridges may create an episode of multi-continental droughts in the southern hemisphere’s subtropics^32^.

### ENSO driving drought-rich periods over subtropical regions of the Southern Hemisphere

Many atmospheric and coupled land–atmosphere processes are responsible for drought events, although one can attribute large-scale drought events over different continents to anomalous SSTs over different oceanic basins^42,62–64^. Significantly, in response to the patterns of SST anomaly^42,65^, large-scale teleconnections may develop as part of the planetary-scale feedback involving the atmosphere and ocean, simultaneously affecting precipitation and temperature distribution over different continents.

**Fig. 4:**
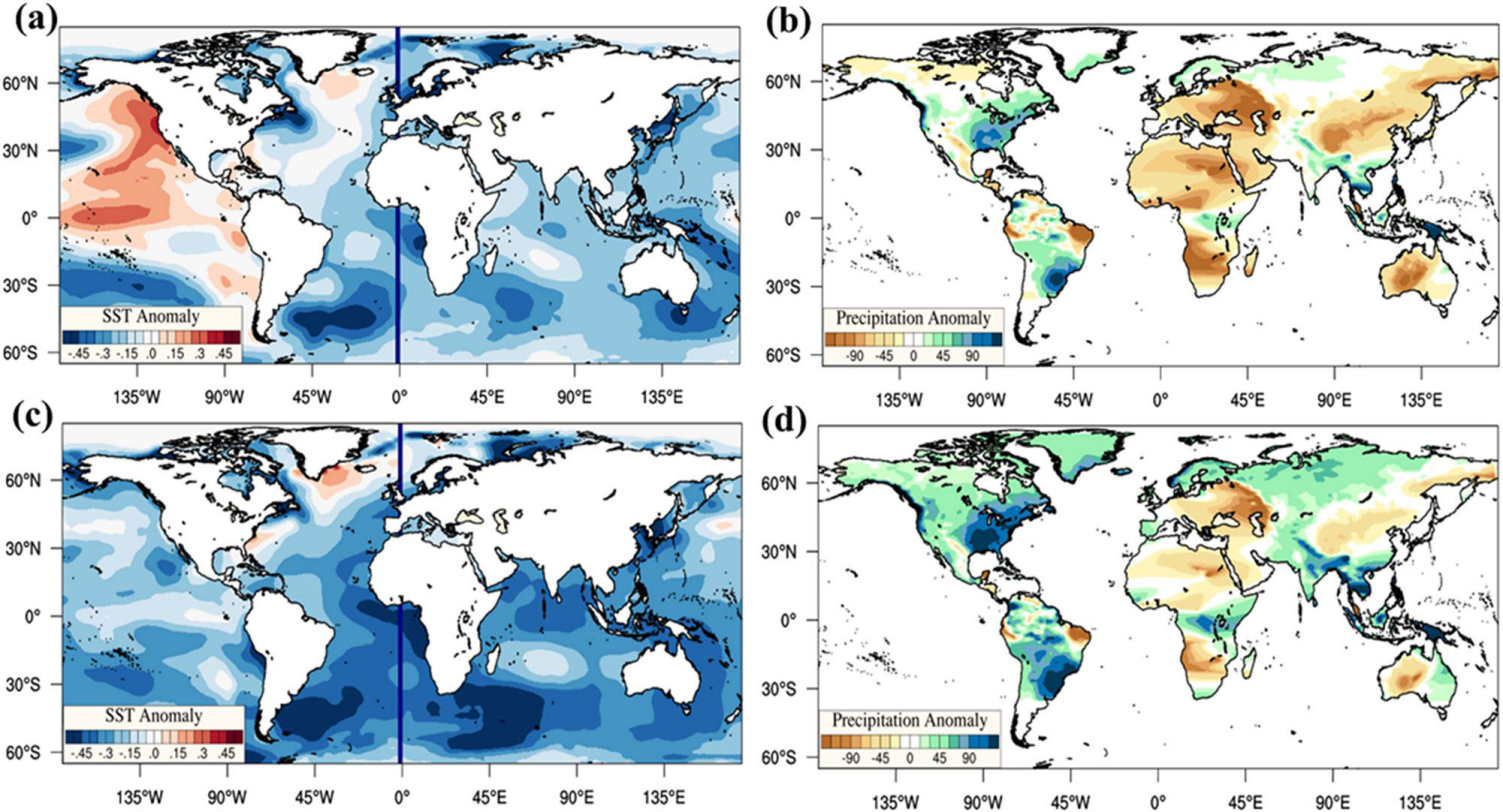
El Nino Southern Oscillations (ENSO) driving drought-rich periods over subtropical regions of southern hemisphere. **a**, **c** Sea surface temperature (SST) anomaly **b**, **d** Precipitation anomaly when (**a**, **b**) drought is occurring in both (~AND logical condition) of the chosen regions and drought is occurring in at least (~OR logical condition) one of the chosen regions (**c**, **d**). The anomaly of precipitation and SST corresponding to the ‘AND condition’ the ‘OR condition’ is deseasonalized for suitable interpretation. Positive anomaly extended from eastern to Central Pacific indicates El Nino, which induces precipitation deficit nearly over the whole globe, especially prominent in Northeast Brazil in South America, South of Africa, and North of Australia, indicating possible drought conditions.

To ascertain the physical process driving the inter-continental scale synchronization between Altiplano (Bolivian High) and South Africa regions, we first choose a 2° × 2° box (Fig. 3b, Box F) over South Africa (high CD and average synchronization density). Omnipresent high DC over both boxes (Box E and F in Fig. 3b) also ensures that the occurred multi-continental droughts are regionally extensive over Southern Africa and South America. To distinctively quantify the driving meteorology, we consider conditions when both Box E and Box F (Fig. 3b) experience drought and when drought is dominant in Box E (Fig. 3b) or F (Fig. 3b), denoted as AND and OR conditions, respectively. We identify the months when the percentage area under drought is very high in Box E and F (Fig. 3d). The composite anomaly of precipitation and SST corresponding to the AND condition (Fig. 4a, b) and the OR condition (Fig. 4c, d) is deseasonalized with respect to the long term mean monthly meteorology of each grid location (mean monthly precipitation and SST over the climatological period 1981–2010). We observe a strong positive SST anomaly of 0.6–0.8 °C extending from the eastern Pacific (near northwest of South America) to the central Pacific, with a higher anomaly over the equatorial region (Fig. 4a). This positive anomaly is the indisputable signature of the El Niño condition, the negative phase of the Southern Oscillation where the eastern and central Pacific has a positive SST anomaly.

On closer inspection, spatially synchronized drought (AND condition—both regions going through droughts) occurs typically during strong El Niño episodes (i.e., 2015, 1998, 1995, and 1983 are El Niño years). During such conditions, a substantial precipitation deficit is noticed over Northeast Brazil and South Africa (Fig. 4a, b). However, the slight positive precipitation anomaly over the high altitude Altiplano would have little influence as the residence time of surface water decreases significantly with the increase in average topographic slope. In contrast, the events related to the OR condition (one of the regions is witnessing droughts) typically happen either during the summer of neutral (not El Niño and La Nina) years or in the successive months (or a year) following the El Niño episodes (Fig. 4c, d). For example, during OR conditions, a combination of higher rainfall (Fig. 4d) and reduced rainfall (Fig. 4d) is observed over the selected regions. Thereby, the OR drought events are possibly due to the seasonal behavior of hydroclimatic anomalies. This contrasts with the AND condition where central and eastern Pacific SST anomalies drive synchronized drought over western South America, Northeast Brazil, and South Africa.

Such large-scale SST anomaly induces low pressure and convergent winds in the lower troposphere and divergent winds (high pressure) in the upper troposphere over the Pacific, vitally influencing the planetary circulation at shorter to longer time scales. Furthermore, an increase of upper tropospheric pressure over the Central Pacific affects subtropical Rossby waves, which influence the stationary anticyclones (such as Bolivian or Botswana High) over the continents in the southern hemisphere^32^. These El Niño modulated anticyclones lead to dominant sinking motion, reducing rainfall (Fig. 4d) and contributing to large-scale droughts over a large area stretched throughout the southern hemisphere continents^32,63^. To validate further, we also provide a correlation estimate between the southern oscillation index and the ScPDSI on a global scale (Supplementary Fig. 11a, b). Interestingly, besides El Niño, other teleconnections (i.e., MJO) also influence such synchronous drought occurrences over the continents mentioned above^32^.

### Comparing short and long-term drought events networks

Drought events can occur at seasonal to multiyear timescales, and each event can be associated with different physical mechanisms. Here, we evaluate the dependence of network and synchronization characteristics on drought duration (or persistence) by comparing the analysis of short-term (duration ≤6 months) and long-term (>6 months) drought events. Heterogeneity among the spatial distribution of DC in long-term (Supplementary Fig. 12a) and short-term (Supplementary Fig. 12b) drought events is noticeable. For example, Northwest USA exhibits spatially more pronounced high DC values in short-term drought events, suggesting this region is more likely to synchronize with other grid locations for short-term drought events. The role of soil moisture in propagating droughts from this region has been noted in previous studies^4^. On the other hand, areas with historical episodes of persistent droughts such as South Africa, Australia, the Midwest USA, and the entire Sahel display a very high DC for long-term drought events (Supplementary Fig. 12a). Interestingly, droughts over many of these regions are driven by oceanic SST, as documented in the previous studies^32,66,67^. Overall, the magnitude of DC is comparatively higher for long-term drought events, indicating that more areas (over the globe) will be under drought whenever such extreme events are persistent. We derive a ratio-based metric DC ratio to study the implication of drought durations on the network characteristics. The DC ratio is estimated by taking the logarithm of the ratio between DC values for long-term drought and short-term drought events. A positive value indicates a comparatively higher number of linkages of a grid location when long-term droughts are considered. As expected, strong positive values of the DC ratio are most prominent over eastern and southeastern Australia, Southern Africa, Western India, West Africa, and Midwest USA (Supplementary Fig. 12c).

The latitudinal distribution (longitudinally averaged) of DC for short- and long-term droughts (Supplementary Fig. 12d) further highlights the influence of oceanic SST as long-term drought exhibits higher linkage characteristics over the southern hemisphere, where the ocean’s role is comparatively higher due to the smaller land area. Evidently, long-term droughts are really extensive and possibly more driven by oceanic SST. In contrast, short-term droughts are local process-driven (land-atmospheric processes) and thus cease to spread once the drying condition reverses. However, the distinctive influence of SST and soil moisture on network characteristics of drought events should be further analyzed in future work.

## Discussion

To understand how droughts are synchronized (or teleconnection) globally, we performed a CN analysis of the global drought events for 1901–2018 using the ScPDSI at a spatial resolution of 0.5° × 0.5°. We aim to reveal the synchronization structure of drought events and develop a topological understanding (globally) using the network coefficients. We observe that droughts have a highly heterogeneous linkage structure with few locations that are rich in connections surrounded by areas with fewer linkages. We call these rich, affluent locations ‘drought hubs’. The CN analysis further quantitatively explains the ‘rich club phenomenon of droughts,’ where drought hubs are located over different continents and display affinity (or connectivity) towards experiencing simultaneous drought conditions, leading to multi-continental drought events. Furthermore, heavy tail characteristics of the degree distribution indicate the scale-free nature of global drought synchronization. The scale-free networks display embedded structural properties such as self-similarity and core–periphery structure. Interestingly, we observe a similar core-periphery structure in the spatial synchronization of global drought events. This aspect of drought synchronization has not been revealed in previous works, but it has important implications for drought prediction and mitigation.

The joint distribution of DC, CC, and MSD reveals a multifold spatial scale associated with drought hubs, possibly distinguishing the spatial scale associated with different teleconnections as well as their compound effects^7^. Major drought hubs are present in the northwestern US, midwestern US, Amazon, the eastern slope of the Andes, South Africa, Arabian deserts, southern Europe, and the Scandinavian region. Furthermore, we test the global drought network for homophily using the concept of assortativity. The positive value of the AC validates that global drought does not form a random network^68^ and the network’s hubs have a substantial affinity towards linking with each other. Such configuration leads to simultaneous large-scale droughts over multiple continents. Focusing on Altiplano (climatological location of the Bolivian High), droughts over this region display substantial synchrony with drought events over Northeast Brazil, South Africa, and Northern Australia (Fig. 4d). The global distribution of SST and precipitation anomalies reveals that El Niño is the primary driver behind the large-scale, multi-continental droughts over South America, Africa, and Australia, enabling the highest spatial scale (>10,000 km) of drought synchronization.

Our results can significantly advance the formulation of societal response and drought mitigation measures. For example, we observe that the highest spatial scale of synchronized drought occurrence (drought hubs with the largest spatial scale) is enabled by El Niño. Furthermore, other atmospheric teleconnections (tropical North Atlantic pattern, NAO) and mid-upper tropospheric structures (Rossby waves and stationary anticyclones) may also drive spatially synchronized multi-continental droughts. These results motivate the need to investigate how such spatially synchronized droughts hitting multiple bread baskets may change in the future. Considering the looming climate crisis, delineating the high-risk regions would greatly help shape agricultural adaptation. For example, to mitigate synchronized failure events, crop production variance-reducing strategies may need to be widely implemented to succeed^8^. Furthermore, understanding the risk of synchronized drought may help monitor and sustain the global food-trade network. For instance, if two countries have a crop sharing (or trade) agreement and have a high risk of experiencing spatially synchronized drought, developing a food trade agreement with other countries with less risk or asynchronous drought occurrences spatially would be more sustainable.

Another critical application is to inform model development to improve global climate models in simulating synchronized droughts, possibly through model tuning and evaluation. For example, the spatial connectivity structure of drought derived in the study may be used to understand the relationship between model biases in simulating droughts in different regions. Typical model evaluation methods include comparing spatiotemporal, distributional, or spectral characteristics. In contrast, the CN of drought derived from observations and climate models can provide objective metrics for process or structure-based model evaluation. For example, studies^69^ have shown that network metrics provide stronger relationships for constraining precipitation projections under climate change than traditional evaluation metrics for storm tracks or precipitation.

The current study has a few limitations, such as using a fixed value of maximum lag time (3 months) between the synchronization of drought events. Although we provide additional results with a 6-month of maximum time lag, future studies should focus on determining the potential influence of multi-scale lag time on the synchronization of droughts. In addition, data length can be a significant limitation (especially for drought-related studies). Furthermore, the ScPDSI typically has a very long tail, with strong inter-monthly and seasonal persistence. Quantifying its influence on the spatial structure of drought events is left for future work. It would also be interesting in the future to quantify the relative influence of general circulation, land surface processes, and anthropogenic water use on shaping the synchronization structure of global drought events.

## Methods

### Data

The ScPDSI data (1901–2018) available at 0.5º × 0.5º spatial resolution was obtained from the Climatic Research Unit (CRU)^8^. Here, the potential evapotranspiration (PET) was calculated using Penman–Monteith, a much more physically based PET method that incorporates information on radiation, humidity, wind speed, and vegetation resistance instead of just temperature^70^. The ScPDSI has been very popular in the research community and has been used in the spatiotemporal investigation of drought and numerous drought impact studies on crops, vegetation, and ecosystems^71,72^. Further, ScPDSI is also a popular indicator for global-scale drought analysis under global warming^14^. The scPDSI index classifies the drought conditions on a scale from −4 for extremely dry conditions to +4 for too-wet conditions. As such, scPDSI is a convenient indicator of the relative extent, spatial location, and severity of drought conditions^70^.

The meteorological data (ocean surface skin temperature) is obtained from NOAA reconstructed SST V5 available at https://psl.noaa.gov/data/gridded/data.noaa.ersst.v5.html. The precipitation is obtained from the Global Precipitation Climatological Center (GPCC) for its observed accuracy and high station density^73^. The precipitation data is downloaded from the following source: https://psl.noaa.gov/data/gridded/data.gpcc.html.

### Drought characteristics

We use the threshold level method^1,3,74^ to determine a drought event’s onset and termination. A drought starts when the ScPDSI is below a predefined threshold. The drought event continues until the threshold gets exceeded. Hence, one can characterize each drought event by its onset, termination, and duration. In the current study, our primary focus is to understand moderate drought over the whole globe. We choose moderate drought as extreme droughts of longer duration generally lack sufficient sample space due to their rare occurrences^3^. Following this scheme, we define an onset of drought when ScPDSI decreases below −2. The drought sustains as long as the ScPDSI value stays below −2.

### Event synchronizations

We apply CN analysis using event synchronization^26,75^ on the binary time series of drought onset events for each grid location over the whole globe. By binary, we imply that only the month with a drought onset is ‘1’ and all others as ‘0’. It is important to note here that only the months with a drought onset have been analyzed.

Previous studies have formulated several techniques to quantify synchronization measures, such as methods based on extracting phase difference using Hilbert transform^76,77^, recurrence of extreme events^78^, and simultaneous occurrence of extreme events^27,75,79^. Recently, non-linear and nonparametric Event Synchronization has become very popular due to its simplicity and computational efficiency, especially in climate sciences. Previous studies have employed ES to understand synchronicity in extreme rainfall^27,79^, heatwave^25^, and regional drought^3^.

The employed method of event synchronization^80^ is briefly illustrated here. For any two grid locations *i* and *j*, ES measures the magnitude of synchronization by counting the number of temporally coinciding drought onsets after allowing a possibility of a dynamic delay between them.

Let us assume that an event occurs at time ${t}_{l}^{i}$ at grid point *i* and ${t}_{m}^{j}$ at grid point *j* (*l* = 1,2,...,*s*_*i*_, *m* = 1,2,…,*s*_*j*_) within a variable time lag ${\tau }_{lm}^{ij}$. This variable time lag is defined as follows:

1 $${\tau }_{lm}^{ij}=\,\min \{\frac{{{t}_{l+1}}^{i}-{{t}_{l}}^{i},{{t}_{l}}^{i}-{{t}_{l-1}}^{i},{{t}_{m+1}}^{\;j}-{{t}_{m}}^{j},{{t}_{m}}^{j}-{t}_{m-1}^{\;j}}{2}\}$$

*s*_*i*_ and *s*_*j*_ are the total numbers of such events at locations *i* and *j*, respectively. The variable time lag is extremely useful for separating drought onsets, avoiding double-counting, and incorporating the obvious stochasticity in extreme events. We define *c*(*i*| *j*), and *c*(* j*|*i*) as the number of times an event appears at location *i* after it appears at location *j* and vice versa, respectively. Hence,

2 $$c(i|j)=\mathop{\sum }\limits_{l=1}^{{s}_{i}}\mathop{\sum }\limits_{m=1}^{{s}_{j}}{J}_{ij}$$

where

$${J}_{ij}=\left\{\begin{array}{ll}1 & {{{{{\rm{if}}}}}}\,0 < {t}_{l}^{i}-{t}_{m}^{j} < {\tau }_{lm}^{ij}\\ 0.50 & \,{{{{{\rm{if}}}}}}\,{t}_{l}^{i}={t}_{m}^{j}\\ 0 & \,{{{{{\rm{if}}}}}}\,{{{{{\rm{otherwise}}}}}}\end{array}\right.$$

similarly, we can define *c*(*j*|*i*). Now we define

3 $${Q}_{ij}=\frac{c(i|\, j)+c( \; j|i)}{\sqrt{{s}_{i}{s}_{j}}}$$

where *Q*_*ij*_ is a measure of the strength of synchronization of drought onsets between grid points *i* and *j* whereas *q*_*ij*_ measures the delay between them. Following Eqs. (2) and (3), *Q*_*ij*_ = 1 indicates complete synchronization between the droughts occurring at *i* and *j*. In the current study, ES has been implemented with a maximum possible time delay (*τ*_max_ = 3 months) of 3 months^26^. Two events will only be considered synchronous if the temporal gap between their occurrences does not exceed 3 months. Once ES computation is completed, we obtained a synchronization matrix with a dimension of 60,787*60,787, where 60,787 is the number of considered grid locations over the globe. The synchronization matrix provides the magnitude of synchronization of drought onsets occurring at any two grid points without considering their direction.

### Derivation of synchronization network

The CN analysis (using ES) is performed following Konapala and Mishra^3^. We construct an undirected network (synchronization network) in the current study. An undirected CN consists of all the nodes (here, grid locations) and a set of pairwise relationships among them. Each connection signifies synchronization in drought onsets between the two connected grid locations without presuming any sense of direction.

The synchronization matrix (adjacency matrix of the synchronization network) is symmetric and contains synchronization values for all possible pairs of grid locations. Thus, it has been utilized to develop the undirected CN, assuming that two points are connected only if the synchronization between them belongs to the strongest 0.01% of all the synchronization values. We have chosen such a strong significance level (0.0001) as the total number of connections present is of the order 10^10^. Such high significance would provide us with statistically crucial links as well as a suitable density of connections to form the network. Let us assume the 99.95th quantile of all the non-zero synchronization values is *θ*. Then the synchronization matrix is converted to a binary adjacency matrix in the following way

4 $${A}_{ij}^{Q}=\left\{\begin{array}{cc}1 & {{{{{\rm{if}}}}}}\,{Q}_{ij} > \theta \\ 0 & {{{{{\rm{if}}}}}}\,{{{{{\rm{otherwise}}}}}}\end{array}\right.$$

Clearly, *A*^*Q*^ is symmetric like the synchronization matrix. Here, the *θ* has been found to be equal to 0.51. To test the statistical significance, independent and uniform distribution of events has been used^26^. The test obtains a *P* value of 0.0001 for the obtained *θ* value. Clearly, the developed undirected connections are highly significant (statistically).

From the synchronization network, we derive four network coefficients, namely DC, BC, CC, and MSD. Furthermore, we also formulate two network metrics, connection density and average synchronization density, which have been illustrated thereafter.

### Description of the network coefficients

DC is the most basic CN measure and can be defined as the number of connections a vertex has. Mathematically, the DC of grid point *j* is formulated as

5 $${{{{{\rm{DC}}}}}}_{j}=\frac{\mathop{\sum }\limits_{i=1}^{N-1}{A}_{ij}}{N-1}$$

where *N* is the total number of vertex or grid points present in the CN. A grid point having a higher degree of centrality has a stronger influence on the functionality or vulnerability of the network. For example, in droughts, a region with high DC may synchronize numerous other locations over the whole network and thus exert a substantial influence on the droughts’ initiation and space–time evolution.

The use of distance measures in CN such as ‘median length of links’ or ‘geographical distance’ has been extensive in previous climate-network studies^24,26,75^. It helps understand the mean possible translation of physical signals (through synchronization) communicated inside a dynamical system. However, the amount of synchronization may vary with distance, and excluding the synchronization value would render the distance measure less meaningful.

In the current study, the average or MSD of grid point *j*, SD_*j*_ is mathematically defined as

6 $${{{{{\rm{SD}}}}}}_{j}=\frac{\mathop{\sum }\limits_{i=1}^{N-1}{Q}_{ij}{A}_{ij}^{Q}{d}_{ij}}{\mathop{\sum }\limits_{i=1}^{N-1}{Q}_{ij}{A}_{ij}^{Q}}$$

Average or mean synchronized distance signifies the scale of synchronization a grid point possesses in the study domain.

Betweenness Centrality *BC*_*j*_ of grid point *j* is defined as the summation of the ratio of all shortest paths between all possible pairs of vertices passing through *j* to the total number of shortest paths between them. Mathematically,

7 $${{{{{\rm{BC}}}}}}_{j}=\mathop{\sum}\limits_{j\ne l\ne m\in V}\frac{{\sigma }_{j}(l,m)}{\sigma (l,m)}$$

Where *σ* (*l*,*m*) is the summation of all possible shortest paths between nodes *l* and *m, and σ*_*j*_ (*l*,*m*) indicates the summation of those paths that also pass through *j*. BC measures the extent to which a grid location lies on the shortest path (in the current study, geodesic paths) connecting all other grid locations. Considering the present context, a region with high BC would have considerable leverage on the drought system by controlling a large amount of information flow.

The clustering coefficient of grid point *j*, CC_*j*_ provides the density of the connections between grid points that are synchronized (connected) with grid point *j*. Assuming *k*_*j*_ the number of grid points connected to grid point *j*, the maximum number of possible connections between these grid points are $\frac{{k}_{j}({k}_{j}-1)}{2}$, whereas the number of actual connections present in the CN is *β*. Then CC_*j*_ is mathematically defined as

8 $${{{{{\rm{CC}}}}}}_{j}=\frac{2\beta }{{k}_{j}({k}_{j}-1)}$$

Considering droughts, a neighborhood of high CC values indicates that grids present in that neighborhood would display spatial coherence in occurrences of droughts. However, events occurring in such regions would seldom display synchronization with any geographically distant neighbor. This suggests that the driving meteorological factors causing droughts in such a region may also show the tendency to move over large distances.

The CD of region A with respect to any grid location is defined as the percentage area of region A having a statistically significant relationship with the considered grid location. The mathematical formulation of CD is provided below:

9 $${{{{{\rm{CD}}}}}}_{i}=\tfrac{\mathop{\sum}\limits_{j\in B}{A}_{ij}}{\mathop{\sum}\limits_{j\in B}j}$$

The average synchronization density (ASD) of region B with respect to any grid location is defined as the average synchronization exerted by the region toward the grid location. The formulation of ASD is presented below:

10 $${{{{{\rm{ASD}}}}}}_{i}=\frac{\mathop{\sum}\limits_{j\in B}{Q}_{ij}}{\mathop{\sum}\limits_{j\in B}j}$$

The CD determines the number of connections between a region and grid location, whereas average synchronization distance (ASD) determines the strength of the connection.

The AC quantifies the *homophily* in a CN. In simple words, the coefficient tells us whether nodes in the network tend to link with nodes of similar connectivity. The mathematical formation of AC is provided below

11 $${{{{{\rm{AC}}}}}}=\frac{{M}^{-1}\mathop{\sum}\limits_{i}{j}_{i}{k}_{i}-[{M}^{-1}\mathop{\sum}\limits_{i}0.5\ast ( \; {j}_{i}+{k}_{i})]^{2}}{{M}^{-1}\mathop{\sum}\limits_{i}0.5\ast ({j}_{i}^{2}+{k}_{i}^{2})-[{M}^{-1}\mathop{\sum}\limits_{i}0.5\ast ({j}_{i}+{k}_{i})]^{2}}$$

where *M* is the number of nodes and *J*_*i*_ is the degree of the *j*th node. A positive value of the AC indicates the presence of homophily in the network.

Additionally, we provide a table (Table 1) summarizing the definition and physical interpretation of the employed network coefficient used in this study.

## Supplementary information

Supplementary Information

## Data Availability

ScPDSI data is obtained from the CRU: https://crudata.uea.ac.uk/cru/data/drought. The precipitation data is available at https://psl.noaa.gov/data/gridded/data.gpcc.html. The SST data is obtained from https://psl.noaa.gov/data/gridded/data.noaa.ersst.v5.html.

## Code availability

The code for complex network calculation is available from authors upon request.

## References

[1] Mishra AK, Singh VP (2010). A review of drought concepts. J. Hydrol..

[2] Andreadis KM, Clark EA, Wood AW, Hamlet AF, Lettenmaier DP (2005). Twentieth-century drought in the conterminous United States. J. Hydrometeorol..

[3] Konapala G, Mishra A (2017). Review of complex networks application in hydroclimatic extremes with an implementation to characterize spatio-temporal drought propagation in continental USA. J. Hydrol..

[4] Herrera‐Estrada JE, Satoh Y, Sheffield J (2017). Spatiotemporal dynamics of global drought. Geophys. Res. Lett..

[5] Orth R, O S, Zscheischler J, Mahecha MD, Reichstein M (2022). Contrasting biophysical and societal impacts of hydro-meteorological extremes. Environ. Res. Lett..

[6] Gaupp F, Hall J, Hochrainer-Stigler S, Dadson S (2020). Changing risks of simultaneous global breadbasket failure. Nat. Clim. Change.

[7] Singh J, Ashfaq M, Skinner CB, Anderson WB, Singh D (2021). Amplified risk of spatially compounding droughts during co-occurrences of modes of natural ocean variability. npj Clim. Atmos. Sci..

[8] Mehrabi Z, Ramankutty N (2019). Synchronized failure of global crop production. Nat. Ecol. Evol..

[9] Epule ET, Peng C, Lepage L, Chen Z (2014). The causes, effects and challenges of Sahelian droughts: a critical review. Reg. Environ. Change.

[10] Omelicheva MY (2011). Natural disasters: triggers of political instability?. Int. Interact..

[11] Murray-Tortarolo GN, Salgado MM (2021). Drought as a driver of Mexico-US migration. Clim. Change.

[12] Pedersen J (1995). Drought, migration and population growth in the Sahel: the case of the Malian Gourma: 1900–1991. Popul. Stud..

[13] Dai A (2011). Drought under global warming: a review. WIREs Clim. Change.

[14] Dai A (2013). Increasing drought under global warming in observations and models. Nat. Clim. Change.

[15] Hastenrath S (2006). Circulation and teleconnection mechanisms of Northeast Brazil droughts. Prog. Oceanogr..

[16] Trenberth KE, Branstator GW, Arkin PA (1988). Origins of the 1988 North American drought. Science.

[17] Cook ER, Seager R, Cane MA, Stahle DW (2007). North American drought: reconstructions, causes, and consequences. Earth Sci. Rev..

[18] Oñate-Valdivieso F, Uchuari V, Oñate-Paladines A (2020). Large-scale climate variability patterns and drought: a case of study in South America. Water Resour. Manag..

[19] Donges JF, Petrova I, Loew A, Marwan N, Kurths J (2015). How complex climate networks complement eigen techniques for the statistical analysis of climatological data. Clim. Dyn..

[20] Agarwal, A., Marwan, N., Rathinasamy, M., Merz, B. & Kurths, J. Multi-scale event synchronization analysis for unravelling climate processes: A wavelet-based approach (2019).

[21] Newman MEJ (2006). Modularity and community structure in networks. Proc. Natl Acad. Sci. USA.

[22] Tsonis AA, Roebber PJ (2004). The architecture of the climate network. Physica A.

[23] Tsonis AA, Swanson KL, Roebber PJ (2006). What do networks have to do with climate?. Bull. Am. Meteor. Soc..

[24] Donges JF, Zou Y, Marwan N, Kurths J (2009). Complex networks in climate dynamics. Eur. Phys. J. Spec. Top..

[25] Mondal S, Mishra AK (2021). Complex networks reveal heatwave patterns and propagations over the USA. Geophys. Res. Lett..

[26] Boers N, Bookhagen B, Marwan N, Kurths J, Marengo J (2013). Complex networks identify spatial patterns of extreme rainfall events of the South American Monsoon System. Geophys. Res. Lett..

[27] Mondal S, Mishra AK, Leung LR (2020). Spatiotemporal characteristics and propagation of summer extreme precipitation events over United States: a complex network analysis. Geophys. Res. Lett..

[28] Olivares, T., Royo, F. & Ortiz, A. M. An experimental testbed for smart cities applications. in *Proceedings of the 11th ACM international symposium on Mobility management and wireless access* 115–118 (Association for Computing Machinery, 2013). 10.1145/2508222.2508243.

[29] Ozturk U (2018). Complex networks for tracking extreme rainfall during typhoons. Chaos.

[30] Pazouki S, Haghifam M-R, Moser A (2014). Uncertainty modeling in optimal operation of energy hub in presence of wind, storage and demand response. Int. J. Electr. Power Energy Syst..

[31] Pei S, Makse HA (2013). Spreading dynamics in complex networks. J. Stat. Mech..

[32] Reason CJC (2016). The Bolivian, Botswana, and Bilybara Highs and Southern Hemisphere drought/floods. Geophys. Res. Lett..

[33] Konapala G, Mondal S, Mishra A (2022). Quantifying spatial drought propagation potential in North America using complex network theory. Water Resour. Res..

[34] Piechota TC, Dracup JA (1996). Drought and regional hydrologic variation in the United States: associations with the El Niño-southern oscillation. Water Resour. Res..

[35] Teng H, Branstator G, Tawfik AB, Callaghan P (2019). Circumglobal response to prescribed soil moisture over North America. J. Clim..

[36] Hoerling M, Kumar A (2003). The perfect ocean for drought. Science.

[37] Williams AP (2017). The 2016 Southeastern U.S. drought: an extreme departure from centennial wetting and cooling. J. Geophys. Res..

[38] Masih I, Maskey S, Mussá FEF, Trambauer P (2014). A review of droughts on the African continent: a geospatial and long-term perspective. Hydrol. Earth Syst. Sci..

[39] Shanahan TM (2009). Atlantic forcing of persistent drought in West Africa. Science.

[40] Ionita M, Lohmann G, Rimbu N, Chelcea S, Dima M (2012). Interannual to decadal summer drought variability over Europe and its relationship to global sea surface temperature. Clim. Dyn..

[41] Li, R. *Tropical-Extratropical Teleconnections and Atmospheric Drivers of European Drought Events*. (University of Oxford, 2018).

[42] Visbeck MH, Hurrell JW, Polvani L, Cullen HM (2001). The North Atlantic Oscillation: past, present, and future. Proc. Natl Acad. Sci. USA.

[43] Boers N (2014). Prediction of extreme floods in the eastern Central Andes based on a complex networks approach. Nat. Commun..

[44] Konapala G, Mishra A (2020). Quantifying climate and catchment control on hydrological drought in the continental United States. Water Resour. Res..

[45] Barlow, M., Cullen, H. & Lyon, B. *Drought in Central and Southwest Asia: La Niña, the Warm Pool, and Indian Ocean Precipitation*. 10.1175/1520-0442(2002)0152.0.CO;2 (2002).

[46] Dijk AIJMV (2013). The Millennium Drought in southeast Australia (2001–2009): natural and human causes and implications for water resources, ecosystems, economy, and society. Water Resour. Res..

[47] Sheffield J, Andreadis KM, Wood EF, Lettenmaier DP (2009). Global and continental drought in the second half of the twentieth century: severity–area–duration analysis and temporal variability of large-scale events. J. Clim..

[48] Huang X (2019). Northern Hemisphere land monsoon precipitation changes in the twentieth century revealed by multiple reanalysis datasets. Clim. Dyn..

[49] Luo M, Feng J, Xu Z, Wang Y, Dan L (2019). Evaluating the performance of five twentieth-century reanalysis datasets in reproducing the severe drought in northern China during the 1920s-1930s. Theor. Appl Climatol..

[50] Donges JF, Zou Y, Marwan N, Kurths J (2009). The backbone of the climate network. EPL.

[51] Hoerling M, Hurrell J, Eischeid J, Phillips A (2006). Detection and attribution of twentieth-century northern and southern African rainfall change. J. Clim..

[52] Nnamchi HC, Dike VN, Akinsanola AA, Okoro UK (2021). Leading patterns of the satellite-era summer precipitation over West Africa and associated global teleconnections. Atmos. Res..

[53] Ummenhofer CC, D’Arrigo RD, Anchukaitis K, Buckley BM, Cook ER (2012). Links between Indo-Pacific climate variability and drought in the Monsoon Asia Drought Atlas.

[54] Albert R, Barabási A-L (2002). Statistical mechanics of complex networks. Rev. Mod. Phys..

[55] Watts DJ, Strogatz SH (1998). Collective dynamics of ‘small-world’ networks. Nature.

[56] Li L, Alderson D, Doyle JC, Willinger W (2005). Towards a theory of scale-free graphs: definition, properties, and implications. Internet Math..

[57] Lanckriet S, Frankl A, Adgo E, Termonia P, Nyssen J (2015). Droughts related to quasi-global oscillations: a diagnostic teleconnection analysis in North Ethiopia. Int. J. Climatol..

[58] Berahmand K, Samadi N, Sheikholeslami SM (2018). Effect of rich-club on diffusion in complex networks. Int. J. Mod. Phys. B.

[59] McAuley JJ, da Fontoura Costa L, Caetano TS (2007). Rich-club phenomenon across complex network hierarchies. Appl. Phys. Lett..

[60] Chen, T., Weng, S. & Schubert, S. *Maintenance of Austral Summertime Upper-Tropospheric Circulation over Tropical South America: The Bolivian High–Nordeste Low System*. 10.1175/1520-0469(1999)056<2081:MOASUT>2.0.CO;2 (1999).

[61] Marengo JA, Torres RR, Alves LM (2017). Drought in Northeast Brazil—past, present, and future. Theor. Appl Climatol..

[62] Woollings T, Hannachi A, Hoskins B (2010). Variability of the North Atlantic eddy-driven jet stream. Q. J. R. Meteorol. Soc..

[63] Vicente‐Serrano, S. M. et al. A multiscalar global evaluation of the impact of ENSO on droughts. *J. Geophys. Res.* **116**, D20109 (2011).

[64] Nelson DB (2011). Drought variability in the Pacific Northwest from a 6,000-yr lake sediment record. Proc. Natl Acad. Sci. USA.

[65] Cai W, Whetton PH (2001). Modes of SST variability and the fluctuation of global mean temperature. Clim. Dyn..

[66] Giannini, A., Saravanan, R. & Chang, P. Oceanic forcing of Sahel rainfall on interannual to interdecadal time scales. *Science* **302**, 1027–1030 (2003). 10.1126/science.1089357 14551320

[67] Reason CJC, Rouault M (2002). ENSO-like decadal variability and South African rainfall. Geophys. Res. Lett..

[68] Newman MEJ (2002). Assortative mixing in networks. Phys. Rev. Lett..

[69] Nowack P, Runge J, Eyring V, Haigh JD (2020). Causal networks for climate model evaluation and constrained projections. Nat. Commun..

[70] Schrier G, van der, Barichivich J, Briffa KR, Jones PD (2013). A scPDSI-based global data set of dry and wet spells for 1901–2009. J. Geophys. Res..

[71] Pandžić K (2022). Application of the self-calibrated palmer drought severity index and standardized precipitation index for estimation of drought impact on maize grain yield in Pannonian part of Croatia. Nat. Hazards.

[72] Wang Q (2019). Assessing the impacts of drought on grassland net primary production at the global scale. Sci. Rep..

[73] Sun Q (2018). A Review of Global Precipitation Data Sets: Data Sources, Estimation, and Intercomparisons. Rev. Geophysics.

[74] Yevjevich, V. M. *Objective Approach to Definitions and Investigations of Continental Hydrologic Droughts*. (1966).

[75] Malik N, Bookhagen B, Marwan N, Kurths J (2012). Analysis of spatial and temporal extreme monsoonal rainfall over South Asia using complex networks. Clim. Dyn..

[76] Rosenblum MG, Pikovsky AS, Kurths J (1997). From phase to lag synchronization in coupled chaotic oscillators. Phys. Rev. Lett..

[77] Schiff SJ, So P, Chang T, Burke RE, Sauer T (1996). Detecting dynamical interdependence and generalized synchrony through mutual prediction in a neural ensemble. Phys. Rev. E.

[78] Marwan N, Carmen Romano M, Thiel M, Kurths J (2007). Recurrence plots for the analysis of complex systems. Phys. Rep..

[79] Boers N (2019). Complex networks reveal global pattern of extreme-rainfall teleconnections. Nature.

[80] Quian Quiroga R, Kreuz T, Grassberger P (2002). Event synchronization: a simple and fast method to measure synchronicity and time delay patterns. Phys. Rev. E.

