# Supplementary material for: Global droughts connected by linkages between drought hubs
Source: Nat Commun. 2023 Jan 10;14:144. doi: 10.1038/s41467-022-35531-8 (PMC9832160; doi:10.1038/s41467-022-35531-8)
Supplement: Supplementary file 1 — Supplementary Information [file 41467_2022_35531_MOESM1_ESM.docx]

**Supplementary Information**

**Increase in Co-Evolution of Global Droughts is Controlled by Drought-hubs**

**Somnath Mondal^1^, Ashok Kumar Mishra^1^, Ruby Leung^2^, Benjamin Cook^3,4^**

^1^Glenn Department of Civil Engineering,

Clemson University, South Carolina, USA

^2^Atmospheric Sciences and Global Change Division,

Pacific Northwest National Laboratory, Richland, Washington, USA

^3^NASA Goddard Institute for Space Studies, New York, NY, USA,

^4^Lamont-Doherty Earth Observatory, Columbia University, Palisades, NY, USA


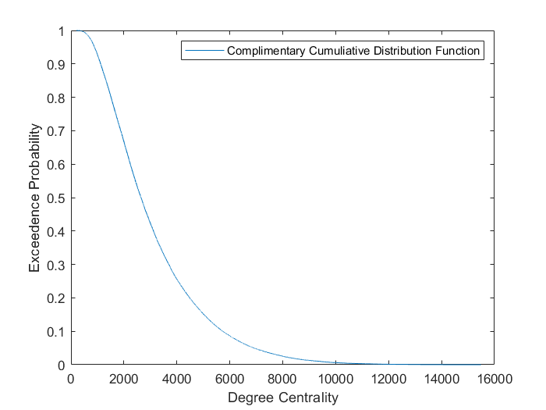


**Supplementary Figure 1.** Complementary Cumulative Distribution Function (CDF) of the degree distribution of global drought. The heavy tail indicates the presence of scale-free characteristics in the higher-order structures of the network.


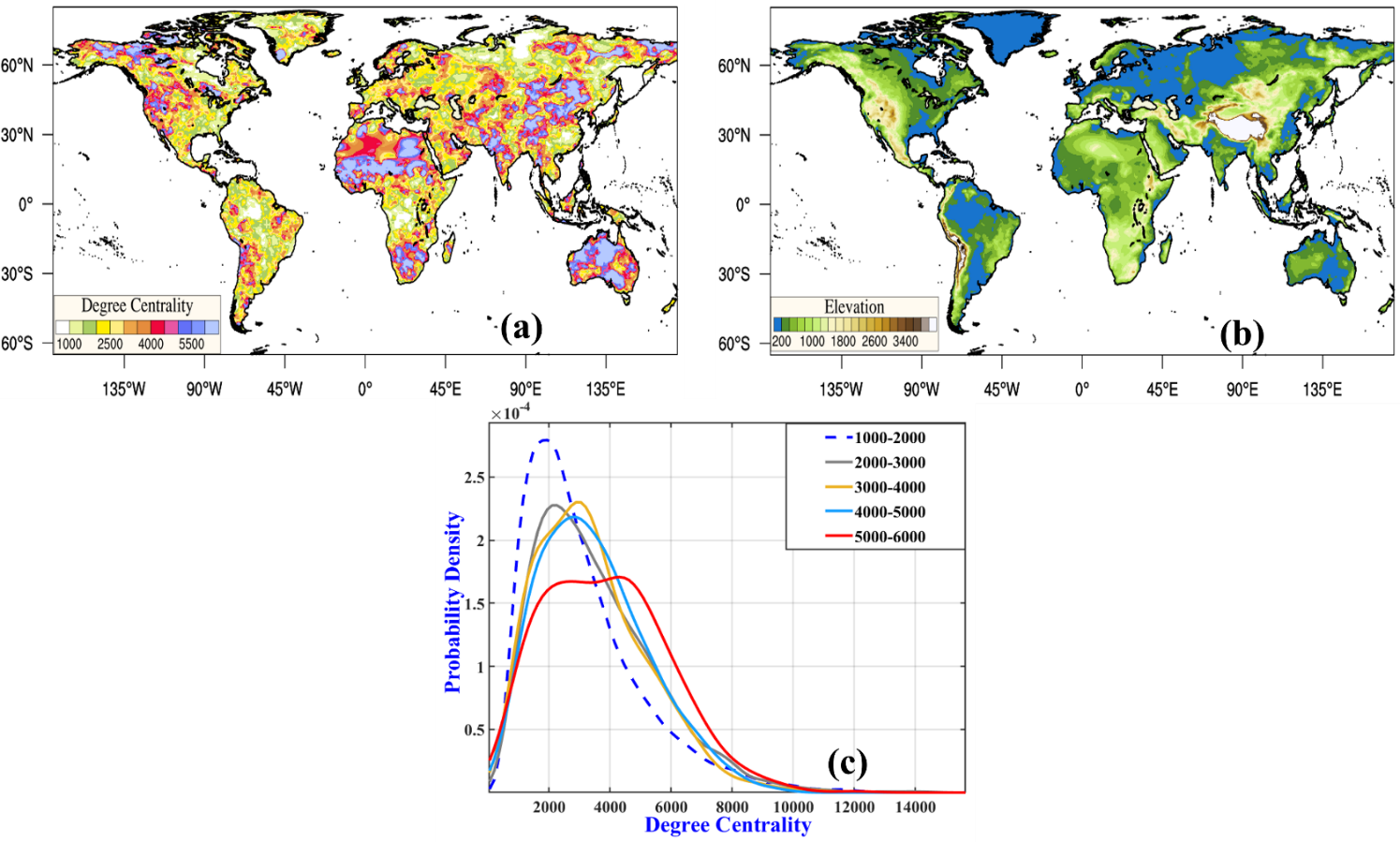


**Supplementary Figure 2.** (a) Spatial Distribution of degree centrality of the drought synchronization network, (b) spatial map of global topography and elevation higher than 4000 m is colored in yellow, and (c) Kernel density estimate of the degree centralities for different elevation bands listed in the legend.

**Supplementary Figure 3.** Spatial Distribution of the degree centrality obtained from the synchronization network of drought events for the period (a) 1901-1958, (b) 1959-2015.


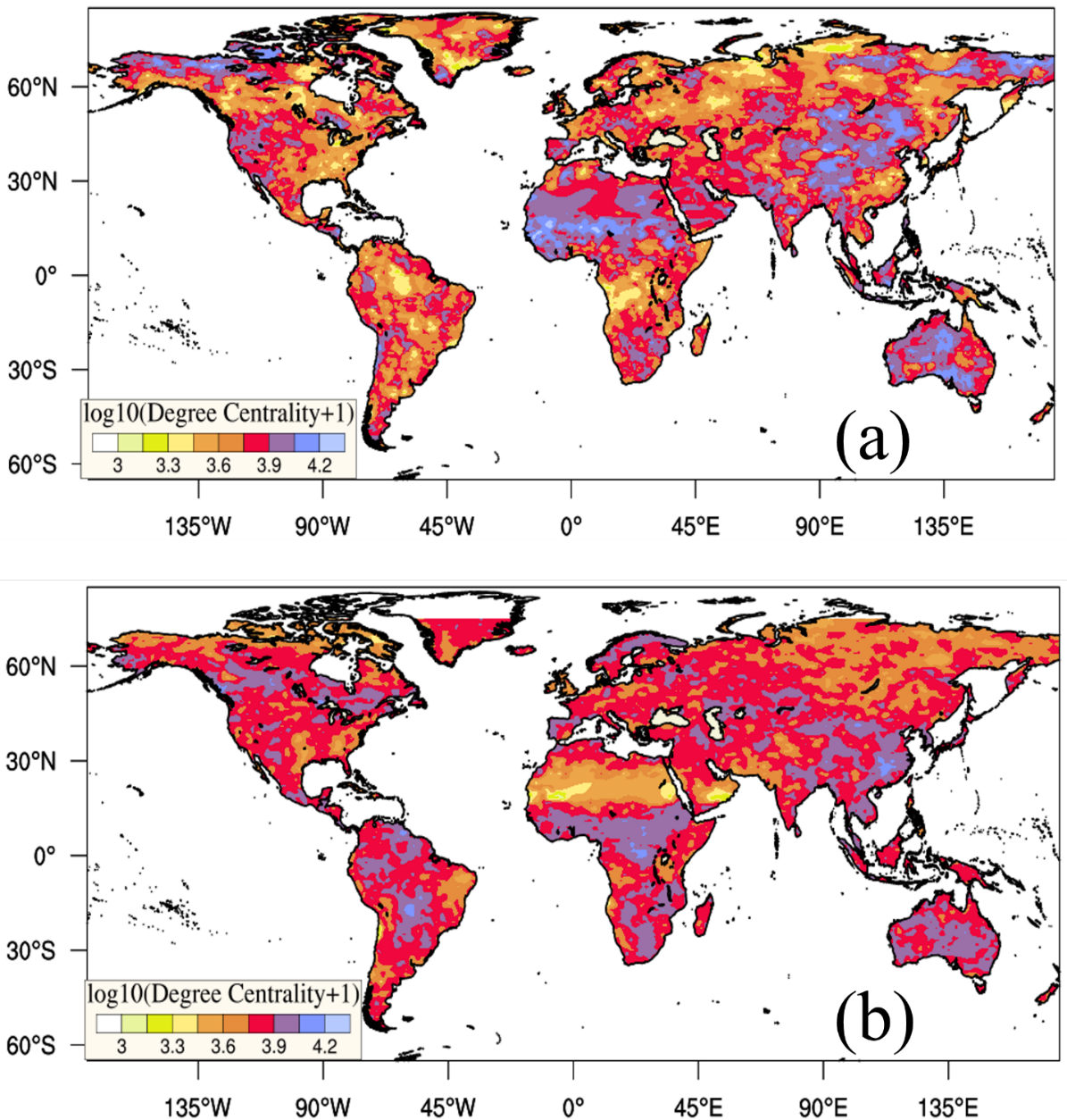


**Supplementary Figure 4**. Spatial Distribution of the degree centrality obtained from the synchronization network of drought events derived from (a) Self-calibrated Palmer Drought Severity Index (ScPDSI) and (b) ERA-20c soil moisture.

**Supplementary Figure 5.** (a) Spatial Distribution of the degree centrality obtained from the synchronization network of drought events with a maximum time lag of 6 months, (b) Scatterplot of degree centrality with a maximum time lag of 3 months (x-axis) and 6 months (y-axis), estimated from the corresponding drought synchronization network. The substantial similarity and spatial correlation indicate that the spatial structure of drought is robust even when a different maximum time lag of synchronization is considered.


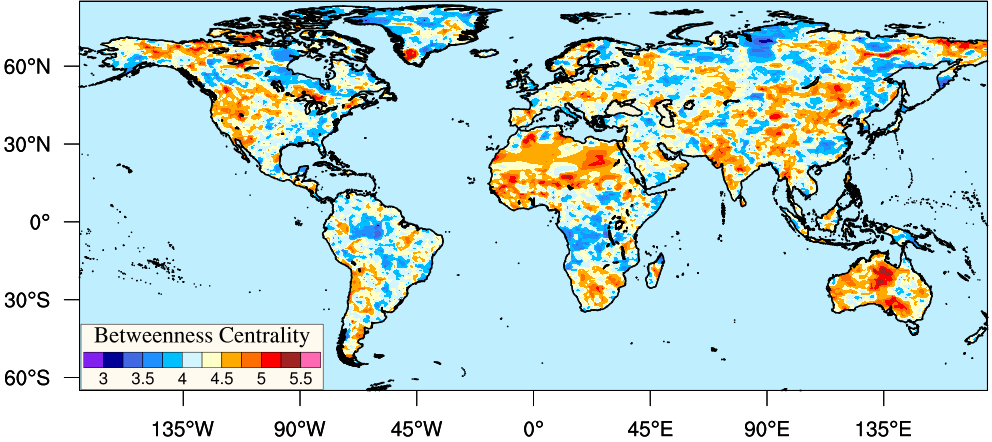


**Supplementary Figure 6.** Betweenness Centrality of the synchronization network of global drought.


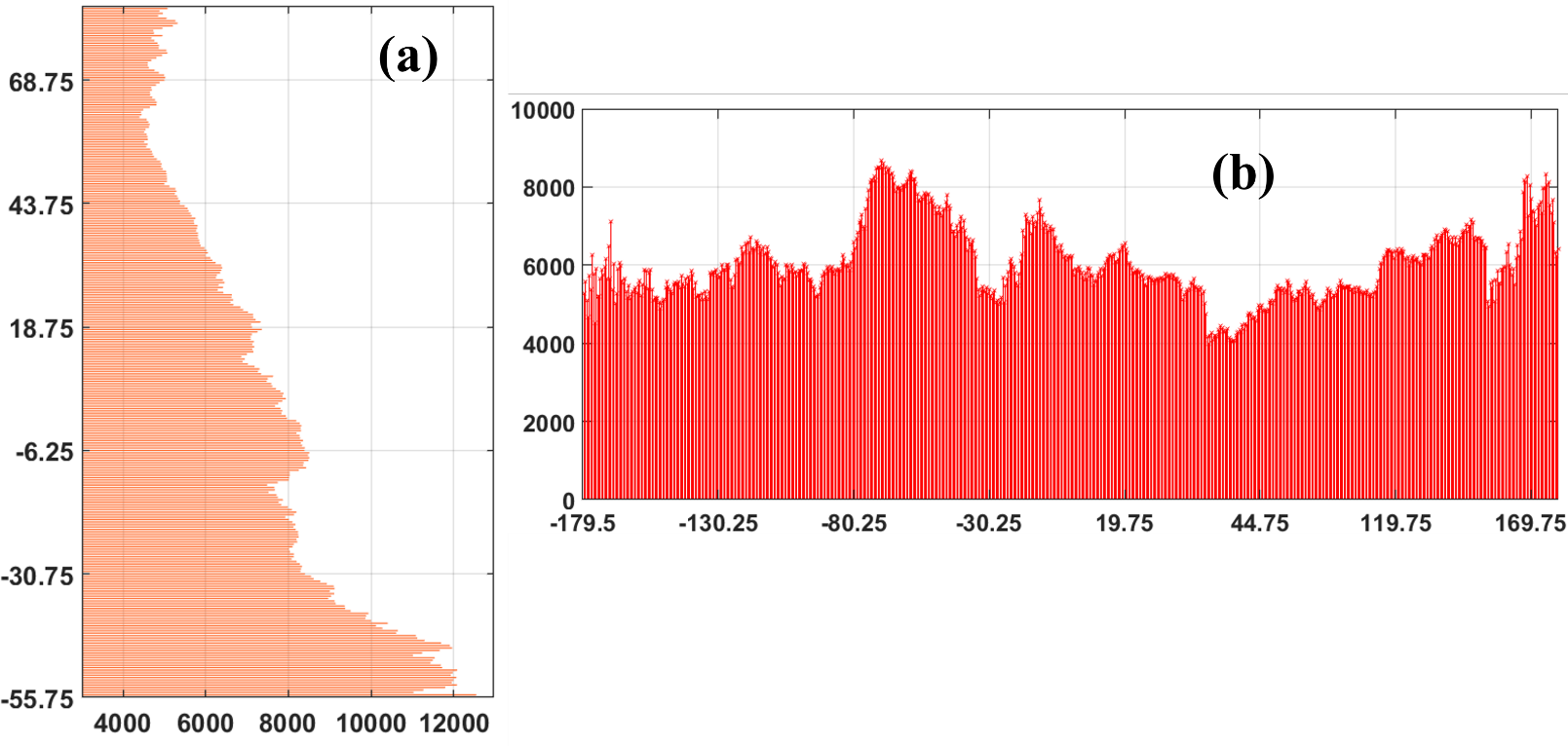


**Supplementary Figure 7.** (a) Latitudinal, (b) Longitudinal mean distribution of Mean Synchronization Distance over the whole globe.


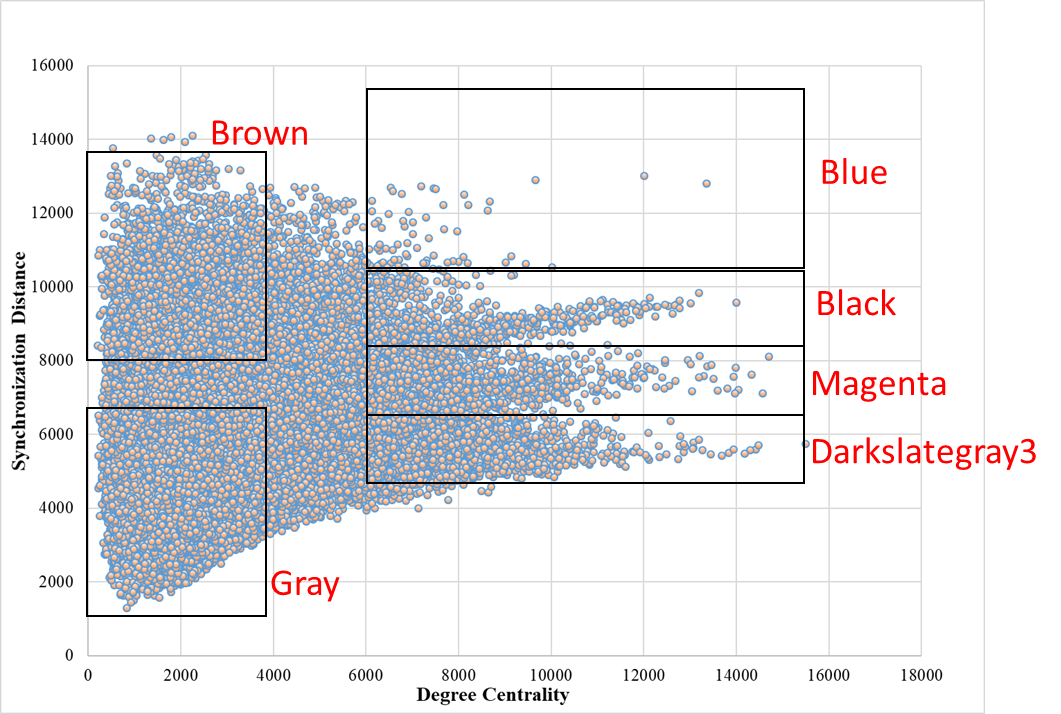


**Supplementary Figure 8.** A qualitative categorization of mean synchronization distance varying on the different spatial scales (Vs Degree Centrality) with the geolocations corresponding to the spatial scale plotted in the same color (Supplementary Figure 10) as mentioned above.


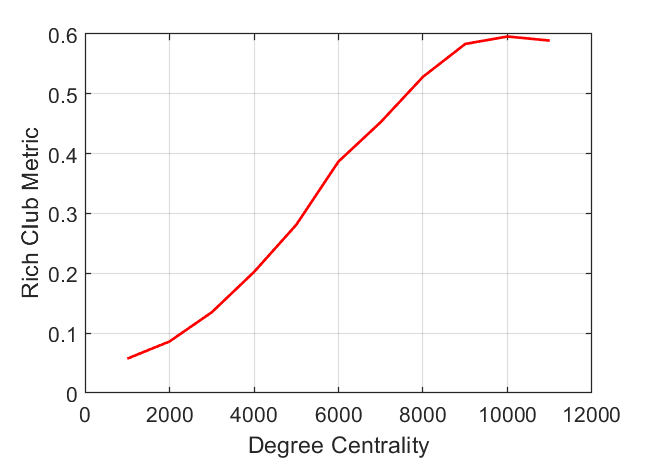


**Supplementary Figure 9.** Monotonically increasing Rich club metric of global drought indicate the presence of the rich-club phenomenon among the drought hubs leading to synchronized drought occurrence over different parts of the planet.


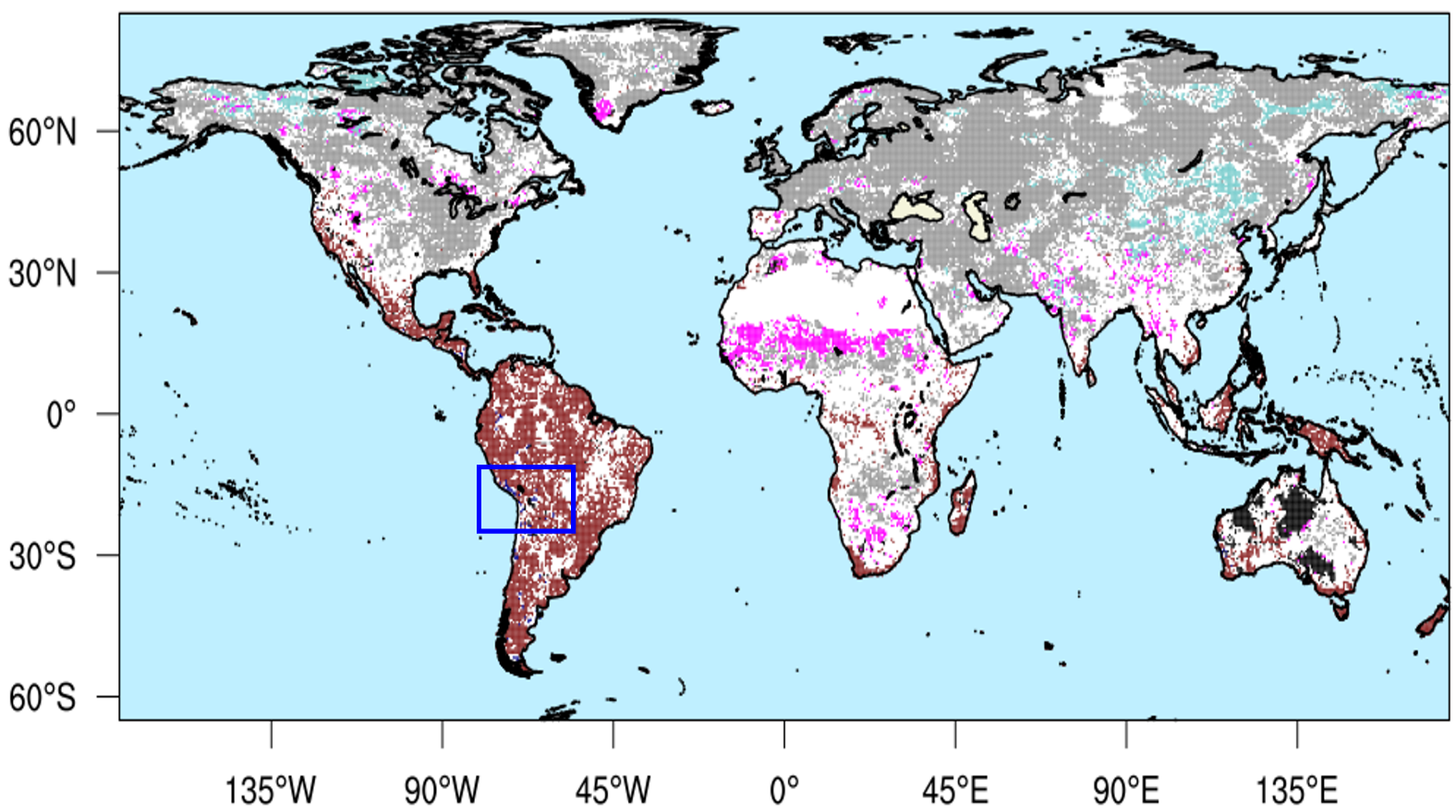


**Supplementary Figure 10.** Grid points are color-coded as per the spatial scale mentioned in Supplementary Figure 8. The blue box indicates the drought hub near altiplano has the highest spatial scale (>10000 Km) and the highest degree centrality.


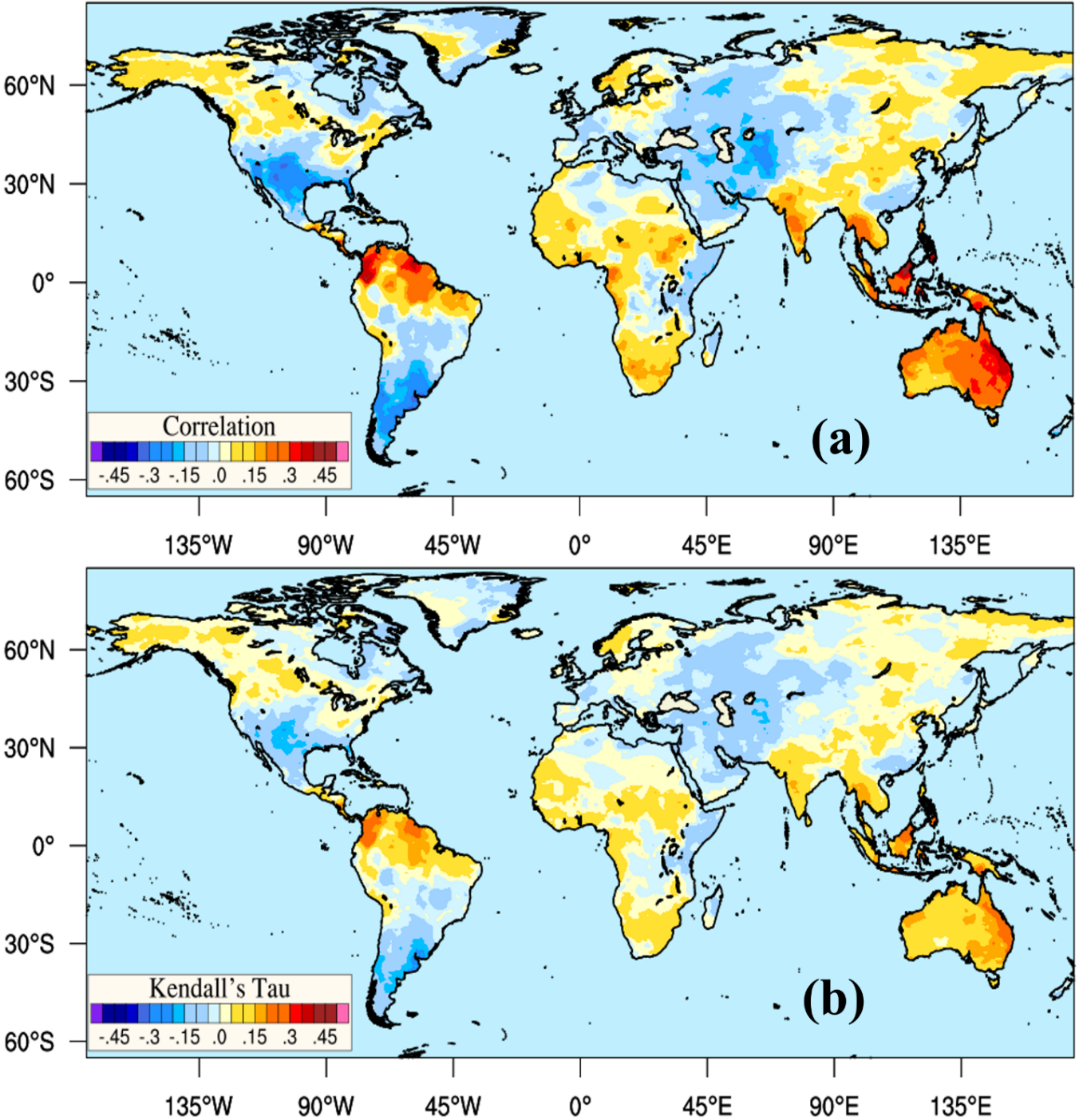


**Supplementary Figure 11.** (a) Pearson’s correlation coefficient, (b) Kendall’s Tau between southern oscillation index and ScPDSI.

**Supplementary Figure 12.** Spatial distribution of degree centrality for (a) long-term drought events (duration>6 months) and (b) short-term drought events (duration<=6 months). (c) The degree centrality ratio is estimated by taking the logarithm of the ratio of degree centrality for long-term drought to that of short-term drought events. Positive (negative) values of the degree centrality ratio indicate that the grid location is more likely to synchronize with other locations when droughts are persistent (short term). (a) the latitudinal mean of degree centrality for long-term (in red color) and short-term (in blue color).
